# Force fields matter in DNA pol η mechanistic analysis

**DOI:** 10.1002/pro.70348

**Published:** 2025-12-23

**Authors:** Reilly Osadchey, Qiang Cui

**Affiliations:** ^1^ Department of Chemistry Boston University Boston Massachusetts USA; ^2^ Department of Physics Boston University Boston Massachusetts USA; ^3^ Department of Biomedical Engineering Boston University Boston Massachusetts USA

**Keywords:** catalysis, DNA polymerase, force field, magnesium, molecular dynamics, QM/MM

## Abstract

DNA polymerase η is of major interest due to debates regarding the role of a third non‐canonical magnesium ion in catalysis. By examining molecular simulations motivated by this mechanistic question and the role of the S113A mutation, we investigate how modern classical force fields compare for treating protein‐DNA complexes and divalent metal ions in enzyme active sites. We find that while the CHARMM36m protein force field is robust, the nucleic acid components are unstable and too flexible, leading to the active site conformation in the reactant state not being well maintained. The addition of the third magnesium ion reduces but does not eliminate these issues, and the observed trends do not match experimental data and the magnesium ions bind too strongly. The AMBER19 force field leads to more stable nucleic acid components, but the standard 12‐6 magnesium ion parameters do not reproduce experimental results on magnesium binding either. After comparing these models with the 12‐6‐4 ion models from OL15, General Amber force field GAFF‐2, and re‐parametrized m12‐6‐4 set, we find that the m12‐6‐4 model best aligns with experimental evidence and semi‐empirical QM(DFTB3)/MM simulations. With these parameters, we conclude that the third magnesium ion binds transiently to the reactant state and strongly to the product state, stabilizing key active site structural features in both. The S113A mutation predominately disrupts the local water network of the active site.

## 1 INTRODUCTION

**FIGURE 1:**
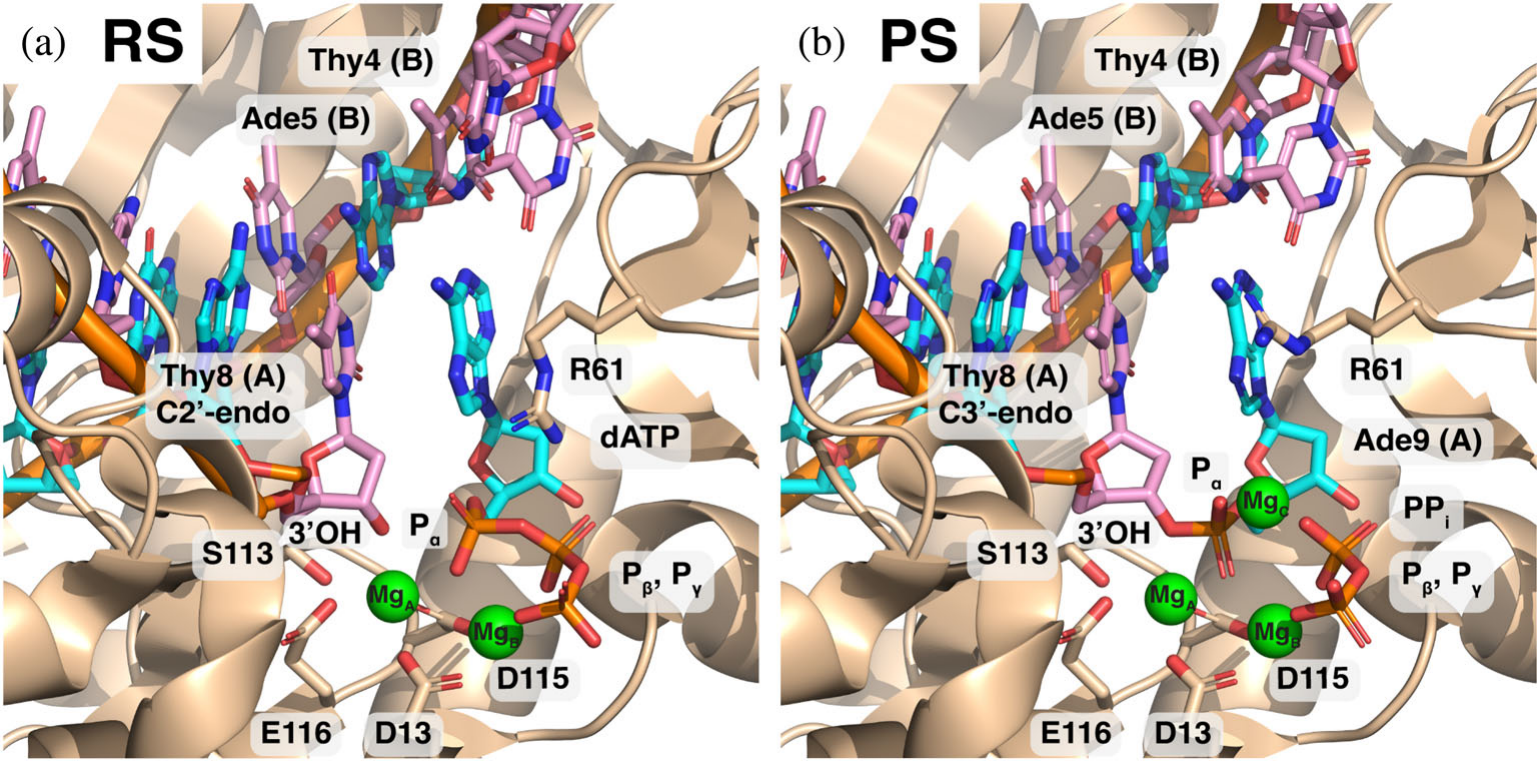
The active site of DNAP in both (a) the reactant state (RS) and (b) the product state (PS), labeled with key residues or atoms that play an important role in the main text (from PDBs 5KFG and 5KFH, respectively (Gao & Yang, 2016)). the DNA is colored by base (purine bases A and G in cyan; pyrimidine bases C and T in pink). Note the alternate conformations of residue R61 in the RS and PS, as well as the terminal end sugar pucker differences between these states.

Deoxyribonucleic acid (DNA) double helices store genetic information of biological systems (Cooper & Hausman, 2015; Garrett & Grisham, 2012). DNA is duplicated by polymerases, which add nucleotide triphosphates (NTP) to growing strands, relying on the other strand as a template (Raper et al., 2018). DNA Polymerase η (referred to as DNAP hereafter) is a human Y‐family polymerase important in trans‐lesion synthesis of UV damage and DNA‐crosslinking by cancer therapeutics (Berdis, 2009; Cruet‐Hennequart et al., 2010; Feltes & Menck, 2022; Kemp & Sancar, 2012; Lehmann et al., 2007; Raper et al., 2018; Steitz, 1999). Canonically, polymerases have two catalytic divalent ions in the active site (Genna et al., 2018; Raper et al., 2018; Steitz, 1999), but time‐resolved x‐ray crystallography shows a transient third magnesium ion, Mg_C_, that appears along with the product state and is thought to be required for catalysis (Chang et al., 2023; Gao & Yang, 2016; Nakamura et al., 2012). There was debate over the role of Mg_C_: does it bind to the reactant state and lower the reaction barrier, bind and stabilize the product state, or even catalyze the reverse reaction? (Chang et al., 2023; Genna et al., 2018; Roston et al., 2019; Wang & Smithline, 2019) X‐ray crystallography of a mismatched nucleotide addition showed that Mg_C_ binds in the reactant state and misincorporation cannot occur without it, suggesting correct nucleotide reaction is too fast to capture Mg_C_ association before the addition step (Chang et al., 2022). Our previous work on DNAP using extensive DFTB3/MM free energy simulations highlighted the role of Mg_C_ in activating an active site water to deprotonate the 3'OH and stabilizing the transition state associated with nucleophilic attack of the phosphate (Roston et al., 2019). Similar conclusion was reached by Stevens & Hammes‐Schiffer, (2018) using DFT/MM based string calculations (Stevens & Hammes‐Schiffer, 2018). We also performed classical molecular dynamics (MD) simulations of DNAP to investigate the impact of the S113A mutation (Gregory et al., 2021). An overview of the active site can be found in Figure 1 with key details labeled as it pertains to later discussions.

Despite the analysis of Gregory et al. (2021), questions remain regarding the effect of the S113A mutation and the reaction mechanism for multiple proton transfer pathways that actives the primer's 3'OH for the nucleophilic attack. To this end, we started by simulating the reactant ternary complex of wild‐type (WT) and S113A mutant of DNAP with two and three magnesium ions to collect multiple well‐equilibrated structures for quantum mechanical/molecular mechanics (QM/MM) simulations. Using the CHARMM36 force field (hereafter referred to as CHARMM), we encountered unexpected base‐pairing behaviors that did not represent experiment well, which motivated further simulations with the AMBER family of force fields. We observed major differences between simulations with the CHARMM and AMBER force fields, and among AMBER simulations with different ion parameterizations. Typical force fields use the Lennard‐Jones functional form with a repulsive $r^{-12}$ distance dependence and an attractive $r^{-6}$ term, while the 12‐6‐4 model adds an attractive $r^{-4}$ term to account for ion‐induced dipole interactions (Li, 2021; Panteva, Giambaşu, & York, 2015a). We further compared these classical simulations to QM/MM simulations using DFTB3 as the QM method and experimental observations as references. We noted differences in active site structure, hydrogen bonding patterns, and ion behaviors that could influence mechanistic interpretations based on MD simulations.

Overall, we recommend using the AMBER force fields for protein‐nucleic acid systems with the 12‐6‐4 ion models (Li et al., 2015a) and specifically the phosphate‐centric re‐parametrized m12‐6‐4 model for ion‐nucleic acid systems (Panteva, Giambasu, & York, 2015b). These results agree the best with experimental observations and DFTB3/MM simulations. With the m12‐6‐4 parameters, the third magnesium ion stabilizes the active site, with transient binding in the reactant state and strong, longer‐lived binding in the product state. The S113A mutation causes disruption of the active site mediated by differences in the local water network of the active site. We also note differences in sugar puckering of the terminal nucleotide between DFTB3 and force fields, concluding that DFTB3's puckering description has room for improvement compared to higher‐level electronic structure calculations. These studies have laid solid groundwork for meaningful QM/MM free energy simulations for DNAP and related protein‐nucleic acid systems.

## 2 RESULTS

In this study, we simulate the WT protein and the mutant S113A, with and without the third Mg_C_ ion, using independent and unbiased classical MD simulations (for a summary of all simulations, see Table 3). These simulations are performed with several force fields to compare their differences, and thus resulting mechanistic interpretations. We also use semi‐empirical DFTB3 QM/MM simulations as a reference where possible. The following results highlight the key force field differences and the key mechanistic differences with respect to the roles of Mg_C_ and the S113A mutation.

We start by comparing the common CHARMM36m (CHARMM) force field (MacKerell et al., 1998, 2004; Vanommeslaeghe et al., 2010) with the Amber ff19SB protein force field (Tian et al., 2020) paired with the OL15 DNA parameters (Galindo‐Murillo et al., 2016)—these are both the newest versions from their family and use the standard 12‐6 Lennard‐Jones (LJ) potentials. The standard divalent ions with Amber are the Li and Merz 12‐6 normal usage set (Li et al., 2013, 2020). For the Amber simulations, the most direct choice would be to treat the dATP ligand as a small molecule using the Generalized Amber Force Field GAFF2 parameters (Wang et al., 2004), which we refer to as the GAFF2 12‐6 simulations. However, there are divalent ions parameters for Amber that use 12‐6‐4 LJ potentials and there are parameters for these ions with the GAFF2‐treated moieties (Li et al., 2015a, 2015b; Li & Merz Jr, 2014) (which we call the GAFF2 12‐6‐4 simulations) as well as the OL15 DNA. To treat the dATP ligand consistently with the OL15 DNA force field divalent parameters, we run what we call the OL15 12‐6‐4 simulations, which treat the dATP as a modified residue from OL15 so we can consistently use the OL15 force field's 12‐6‐4 ion parameters (which was possible for CHARMM simulations). Treating the dATP ligand as part of the OL15 force field also allows us to utilize the Panteva, Giambasu, & York (2015b) m12‐6‐4 ion parameters for the dATP ligand (these parameters correct deficiencies in the OL15 12‐6‐4 parameters) (Panteva, Giambasu, & York, 2015b).

### 2.1 CHARMM shows unstable catalytic center with magnesium rescue while AMBER is stable

**FIGURE 2:**
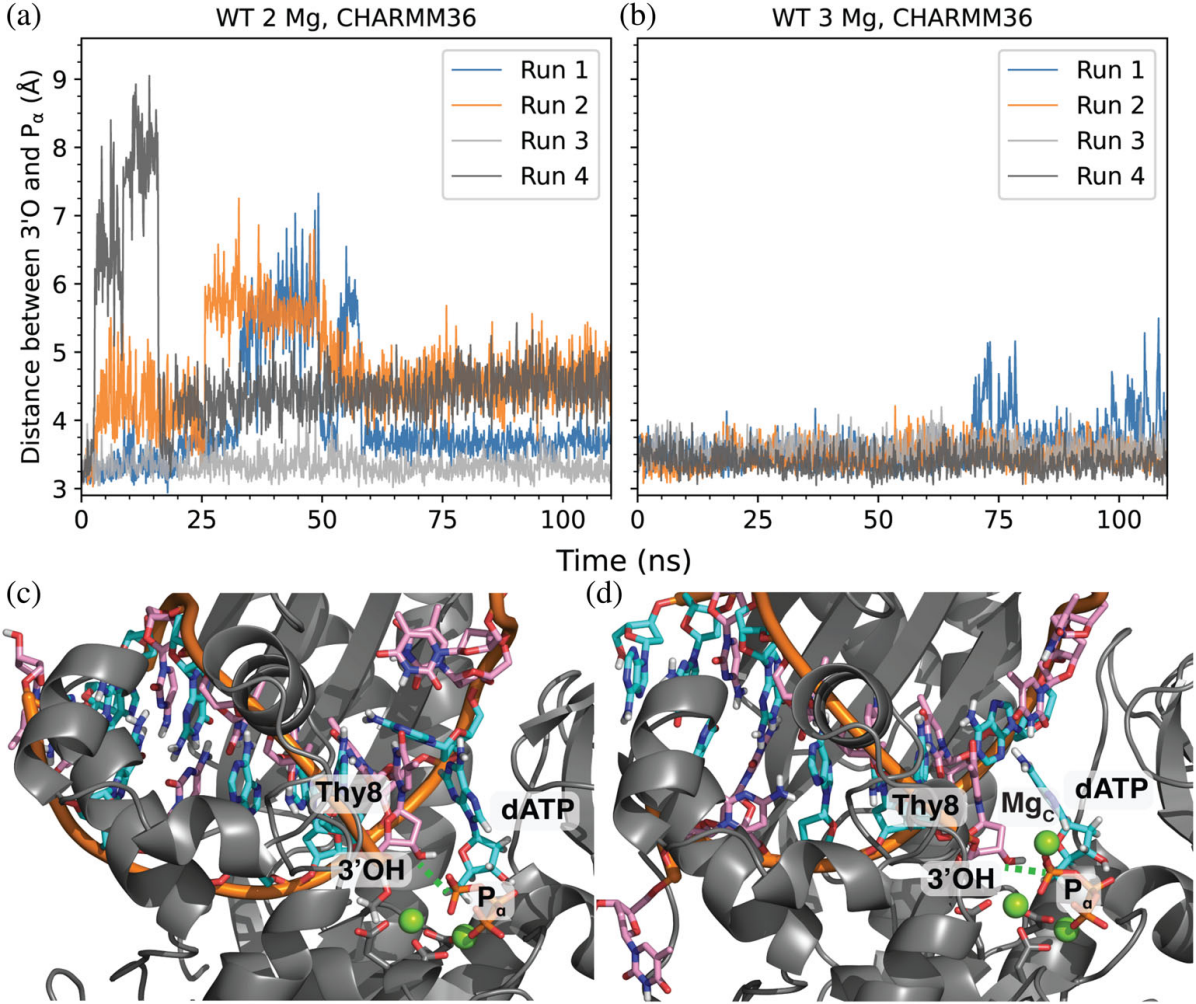
The presence of the third ${\mathrm{Mg}}_{C}$ ion (b) and (d) prevents disruption of DNAP's active site (as measured by the nucleophilic 3'O to $P_{\alpha }$ distance shown as the dashed green line) for the CHARMM force field (a) and (c). Atoms are colored according to: C = cyan (purine bases, A and G) or pink (pyrimidine bases, C and T), H = white, N = blue, O = red, P = orange.

Our MD simulations with the CHARMM36 force field show disruption at the catalytic center when only two magnesium ions are present (Figure 2). Key interactions are broken: the 3' hydroxyl of the terminal Thy (3'OH) no longer directly coordinates to Mg_A_ and is no longer primed for nucleophilic attack on the incoming dATP's α‐phosphate ($P_{\alpha }$). When comparing simulation runs with only two Mg^2+^, this disruption occurs to a greater extent for the S113A mutant than the WT protein (Table 1). In 110 ns simulations, one of the WT runs maintained the aforementioned interactions while none of the S113A mutant proteins did (out of a total of four). The disruption of these interactions occurs concertedly. The WT and S113A mutants have different distance distributions characterizing their final shifted states: the final 3'O to Mg_A_ distance is between 4 and 5 Å for the four shifted runs of the WT protein while only two of the S113A runs end at this distance range. The CHARMM simulations with two Mg^2+^ ions (runs 2 and 4) show the 3'OH hydrogen bonded to the O'4 sugar ring oxygen of the dATP ligand. There is a water in between the 3'O and Mg_A_, but it is hydrogen bonded to S113 due to its coordination geometry. The fact this water cannot hydrogen bond to S113 in the S113A mutant helps explain why this disruption is seen less for the S113A mutant simulations. We have no evidence to suggest this state is catalytically competent considering that the key nucleophilic attack for the reaction is broken. The other two S113A runs end at between 7 and 8 Å. The distance from the 3'O to $P_{\alpha }$ shows that the final distances are smaller for all WT runs than the S113A ones.

**TABLE 1:** Simulations with the CHARMM36 force field show a high number of replicates with disruption of the catalytic center by the reactant nucleotide dATP drifting away from the nucleophilic 3'OH, with the problem being worse for the two Mg ion case, and worse for the S113A mutant (although not at a level of statistical significance).

Protein	Mg^2+^	Drift (No./4)
WT	2	3
S113A	2	4
WT	3	1
S113A	3	2

This disruption is ameliorated by the addition of Mg_C_ (Figure 2). The WT runs had all but one simulation retain the starting contacts between the 3'O and Mg_A_ (Table 1). The run with this interaction broken retained the interaction between the 3'O and $P_{\alpha }$. In the S113A mutant runs, the native conformation was retained in two runs and the two key interactions mentioned above were broken in the other two. In contrast, the AMBER force field shows a stable active site—the dNTP ligand does not shift and maintains key protein contacts in all runs.

**FIGURE 3:**
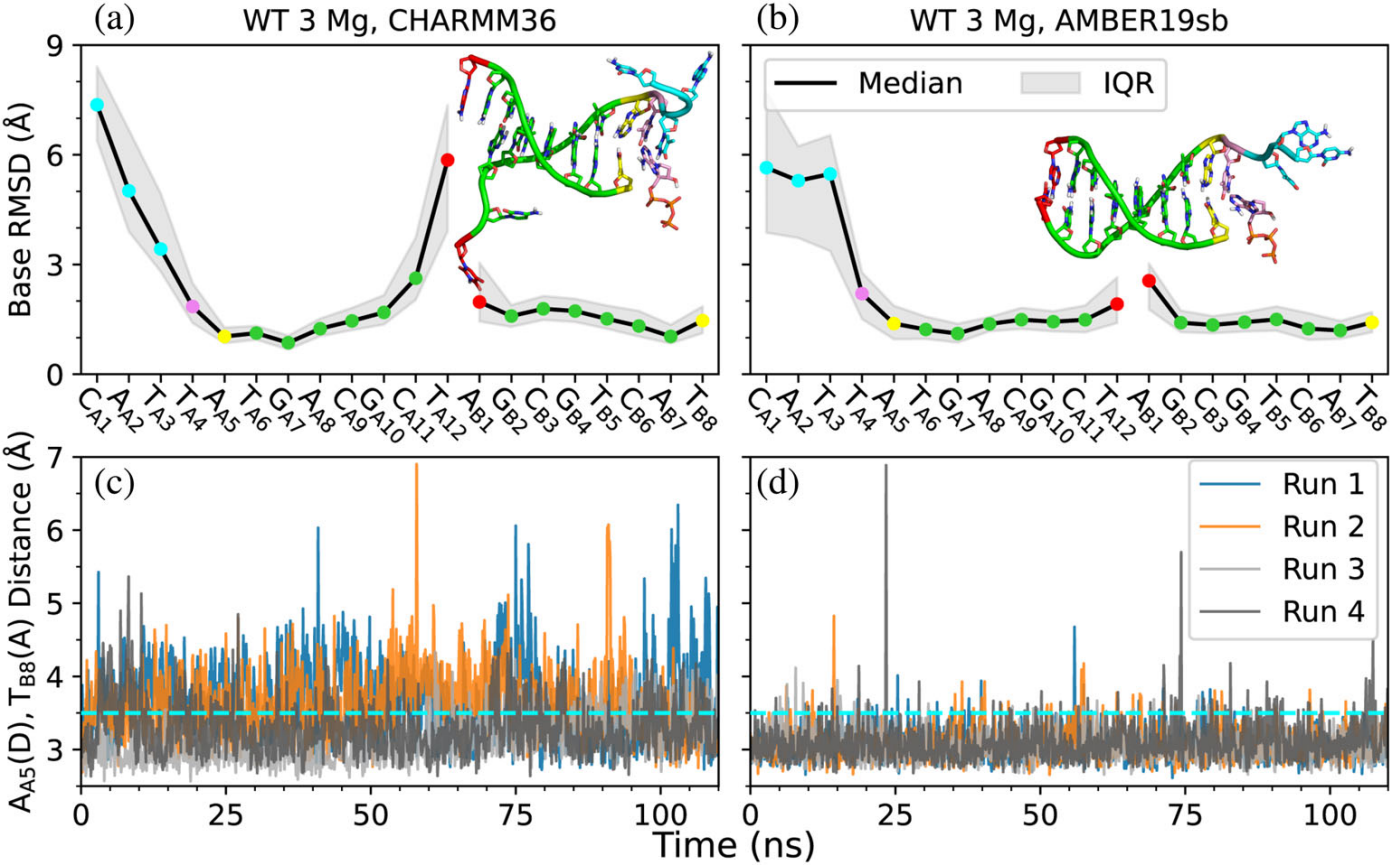
The CHARMM force field produces less stable DNA structures (a) with greater fluctuation in base paring (c) as compared to the AMBER force field (b) and (d). DNA bases are colored based on their positions to help visualize the results. Red: Nonreactive terminal end, green: Non‐terminal and base‐paired, yellow: Active site terminal end, pink: Incoming nucleotide and its base‐pairing partner, cyan: Unpaired. DNA bases are labeled on the upper x‐axes by their identity with DNA strand and base index as subscripts. The blue line in bottom graphs represents our hydrogen bonding donor‐acceptor distance cutoff of 3.5 Å. IQR: Inter‐quartile Range.

Regarding the bound DNA, the CHARMM simulations lead to more flexible structures with larger fluctuations and weaker base pairing than AMBER (Figure 3). The non‐reactive (paired) end of the DNA exhibited large motions that ultimately resulted in the unzipping of the terminal DNA base pairs. This causes higher root mean square deviation (RMSD) values for the next few bases in the DNA strand. The unpaired template strand bases have high RMSD values with both CHARMM and AMBER force fields, but the spread is much less for the CHARMM force field. The base‐pairing residues Thy8 on the growing strand and its complementary base Ade5 (marked in yellow in Figure 3) do not register notable RMSD differences between the two force fields. However, by examining the distance for the Ade5 (donor)–Thy8 (acceptor) hydrogen bond, one sees that this interaction is weaker with the CHARMM force field, as shown by the more frequent break and greater fluctuations. As seen in Figure 2, the configuration from the CHARMM simulation without Mg_C_ shows that the dATP ligand does not form a base pair with the template strand, again reflecting the weak hydrogen bonding between base pairs.

Finally, the CHARMM and AMBER simulations show different binding patterns of Mg_C_ (Table 2). In the CHARMM simulations, all four runs of both the S113A and WT proteins show that Mg_C_ stays bound and coordinated to the $P_{\alpha }$ oxygen where the simulations were started (and the binding mode seen in previous work (Gregory et al., 2021)). This suggests that Mg_C_ binding in the reactant state is strong and necessary for active site organization. Experimentally, Mg_C_ binding is not seen in the reactant state crystal structure—it appears as product forms (Chang et al., 2022; Nakamura et al., 2012). This is likely due to a low population of bound Mg_C_ in the reactant state, given that a Mg_C_‐bound population can be observed when the chemical step is slow due to an incorrect nucleotide substrate (Chang et al., 2022). In contrast to the CHARMM results, the AMBER simulations (GAFF2 12‐6 ions) show that Mg_C_ stays bound in all four of the S113A mutant simulations and none of the WT simulations in the reactant state. The WT protein has Mg_C_ dissociate early in the simulations, either during minimization or early equilibration. These AMBER results suggest that the S113A mutation aids the binding of Mg_C_. Experimental results suggest that the reaction cannot take place without the third Mg ion (Chang et al., 2022; Gao & Yang, 2016), and the reaction can occur in the S113A mutant (Gregory et al., 2021). Therefore, both CHARMM and AMBER simulations with the standard 12‐6 ion model are not congruent with experimental observations concerning Mg_C_ binding. Accordingly, we tested a variety of AMBER 12‐6‐4 ion models to see if they better capture experimental trends.

**TABLE 2:** Binding locations of Mg_C_ in reactant state simulations with different force fields at the start and end of simulation.

Force field^a^	Protein	Runs	Binding to *P*_*α*_ ^b^ start (end)	Binding to $P_{\gamma }$ start (end)
CHARMM36	WT	4	4 (4)	0 (0)
	S113A	4	4 (4)	0 (0)
GAFF2 12‐6	WT	4	0 (0)	0 (0)
	S113A	4	4 (4)	0 (0)
GAFF2 12‐6‐4	WT	5	5 (1)	0 (2)
	S113A	5	2 (0)	0 (1)
OL15 12‐6‐4	WT	5	3 (0)	0 (0)
	S113A	5	5 (0)	0 (0)
m12‐6‐4	WT	5	3 (1)	0 (0)
	S113A	5	2 (1)	0 (0)

*Note*: All starting locations are placed before equilibration at a location representative of the CHARMM36 binding mode. Simulations with the 12‐6‐4 ion parameters were run for 330 ns, three times as long as the 12‐6 simulations at 110 ns.

^a^ Description and references for the force fields. CHARMM36: CHARMM36m (MacKerell et al., 1998; MacKerell et al., 2004; Vanommeslaeghe et al., 2010). GAFF2 12‐6: Amber ff19SB protein (Tian et al., 2020) and OL15 DNA force fields (Galindo‐Murillo et al., 2016) with GAFF2 (Wang et al., 2004) for the dATP ligand and standard divalent Li and Merz 12‐6 normal usage set (Li et al., 2013, 2020). GAFF2 12‐6‐4: Same as GAFF2 except using the 12‐6‐4 ion parameter set (Li et al., 2015a, 2015b; Li & Merz Jr, 2014). OL15 12‐6‐4: Same as above except that the dATP ligand is treated as a modified OL15 DNA residue to ensure it is treated consistently with the other nucleic acids. m12‐6‐4: Same as OL15 12‐6‐4 except with the Panteva, Giambasu, & York (2015b) m12‐6‐4 ion parameters (Panteva, Giambasu, & York, 2015b).

^b^ Some of these runs show unbinding during equilibration, otherwise it occurs during minimization. The GAFF2 12‐6 WT simulations have zero runs bound to $P_{\alpha }$ at the start of production, all of these came unbound during minimization (Figure S.16).

**TABLE 3:** Summary of all unbiased simulations in this work.

Type	Protein	Mg ions	Force field^a^	RS runs^b^	PS runs^b^	Length per run
MM	WT	2	CHARMM36	4	—	110 ns
MM	S113A	2	CHARMM36	4	—	110 ns
MM	WT	3	CHARMM36	4	—	110 ns
MM	S113A	3	CHARMM36	4	—	110 ns
MM	WT	2	AMBER, GAFF2 12‐6	4	5	110, 330 ns
MM	S113A	2	AMBER, GAFF2 12‐6	4	5	110, 330 ns
MM	WT	3	AMBER, GAFF2 12‐6	4	5	110, 330 ns
MM	S113A	3	AMBER, GAFF2 12‐6	4	5	110, 330 ns
MM	WT	2	AMBER, GAFF2 12‐6‐4	5	5	330 ns
MM	S113A	2	AMBER, GAFF2 12‐6‐4	5	5	330 ns
MM	WT	3	AMBER, GAFF2 12‐6‐4	5	5	330 ns
MM	S113A	3	AMBER, GAFF2 12‐6‐4	5	5	330 ns
MM	WT	2	AMBER, OL15 12‐6‐4	5	—	330 ns
MM	S113A	2	AMBER, OL15 12‐6‐4	5	—	330 ns
MM	WT	3	AMBER, OL15 12‐6‐4	5	—	330 ns
MM	S113A	3	AMBER, OL15 12‐6‐4	5	—	330 ns
MM	WT	2	AMBER, OL15 m12‐6‐4	5	5	330 ns
MM	S113A	2	AMBER, OL15 m12‐6‐4	5	5	330 ns
MM	WT	3	AMBER, OL15 m12‐6‐4	5	5	330 ns
MM	S113A	3	AMBER, OL15 m12‐6‐4	5	5	330 ns
QM/MM	WT	2	DFTB3/CHARMM36	5	5	555 ps
QM/MM	S113A	2	DFTB3/CHARMM36	5	5	555 ps
QM/MM	WT	3	DFTB3/CHARMM36	5	5	555 ps
QM/MM	S113A	3	DFTB3/CHARMM36	5	5	555 ps

^a^ In the AMBER simulations, the protein is described with ff19SB (Tian et al., 2020), the DNA with OL15 (Galindo‐Murillo et al., 2016), and the incoming nucleotide with GAFF2 (Wang et al., 2004) or OL15. Several models for the Mg^2+^ are used, as described in detail in Materials and Methods.

^b^ RS, reactant state; PS, product state.

### 2.2 Different AMBER ion models affect Mg_c_ behavior in both reactant and product states

The 12‐6‐4 ion sets tested here (GAFF2, OL15, and the modified OL15 set named m12‐6‐4) all exhibit different behaviors than the AMBER 12‐6 ion model, and there are qualitative differences between their results (Table 2).

The GAFF2 12‐6‐4 ion model retains Mg_C_ binding in all five simulations of the WT protein during equilibration as compared to two of the S113A runs. By the end of 330 ns simulations, only one run maintained the original Mg_C_ binding mode near $P_{\alpha }$ for the WT protein and none for the S113A mutant. Both proteins show Mg_C_ migration to the gamma‐phosphate $P_{\gamma }$ of the incoming nucleotide. There is no crystal structure evidence for this configuration, which is likely to be unproductive for catalysis. The OL15 12‐6‐4 ion model shows, like the GAFF2 12‐6 ion model, that all runs lead to dissociation of Mg_C_ by the end of the 330 ns simulation. The m12‐6‐4 ion model better matches experimental trend in Mg_C_ binding to the protein: the WT and the S113A mutant do not exhibit significant differences in Mg_C_ binding, as is expected. The binding strength seems intermediate (between CHARMM and OL15 12‐6‐4) since two or three runs have Mg_C_ retained at the start of production simulations, and Mg_C_ stayed bound until the end of the 330 ns simulation in only one run (for both WT and S113A).

**FIGURE 4:**
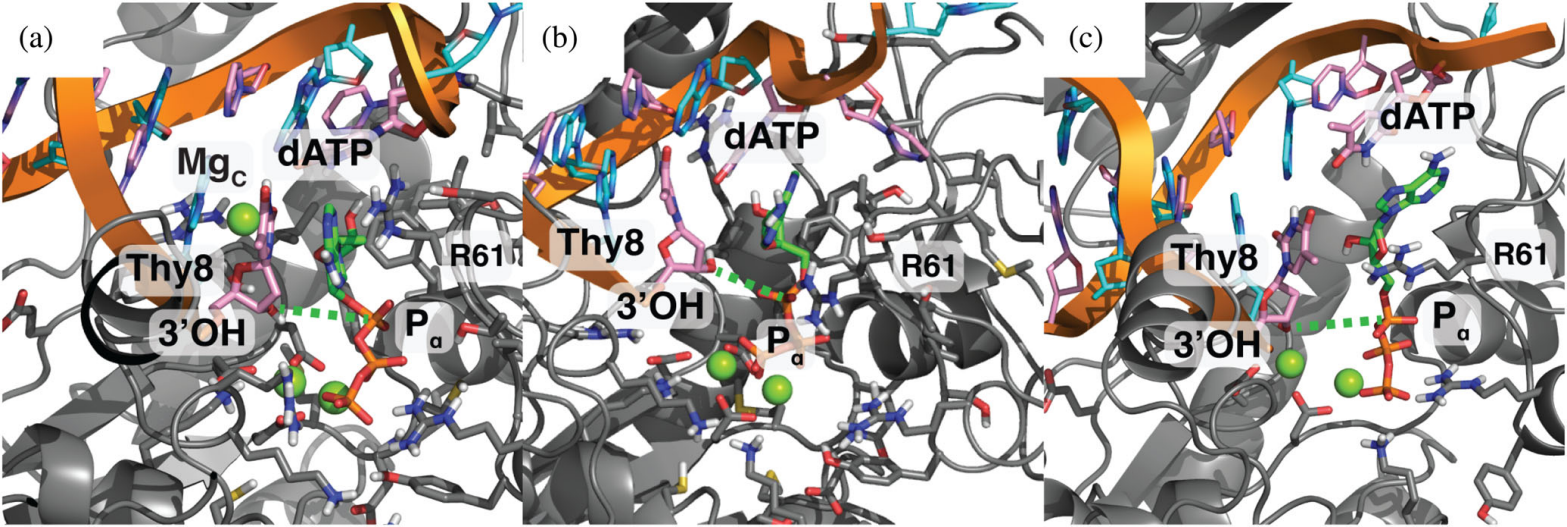
Representative reactant state simulation configurations showing dATP (green carbons) from a simulation of (a) WT m12‐6‐4 and (b) S113A GAFF2 12‐6‐4 as compared to (c) the WT m12‐6‐4 unbinding umbrella sampling simulation (see Figure S.5). In (a) and (b), all protein residues within 5 Å of the dATP ligand are shown. The 3'O to $P_{\alpha }$ distance is shown as the dashed green line.

The catalytic center in the different AMBER 12‐6‐4 ion simulations shows occasional disruption: six simulations out of a total of 60 (Table S.1). These disruptions occur slightly more with the S113A mutant (four) over the WT protein (two). These disruptions occur because base‐pairing between the incoming dATP ligand is broken with the template thymine (Thy4) (Figure 4). There are differences in the specific locations of the base, but all share the sugar repositioning into a water pocket. This disruption separates the nucleophilic 3'OH and Mg_A_, and shows rotation of the dATP ligand triphosphate tail dihedral angles. In the WT m12‐6‐4 configuration shown in Figure 4a, the template strand Thy3 has swung in to interact partially with the growing strand Thy8 and the dATP ligand. In Figure 4b (S113A GAFF2 12‐6‐4 configuration) the dATP ligand is engaged in a base‐stacking configuration with the originally base‐paired Thy4. We wondered whether this could represent on‐pathway unbinding of the dATP ligand (see Figure 4c and additional discussion in the Supplementary Materials).

We also simulated DNAP (WT and the S113A mutant) with two or three Mg^2+^ ions in the product state with most of the AMBER force fields: GAFF2 12‐6, GAFF2 12‐6‐4, and the m12‐6‐4 parameter sets. We simulate both reactant and product states given the debate about whether Mg_C_ binds only to the product state and whether it can catalyze the reverse reaction (Chang et al., 2023). The dATP ligand has been added to the primer strand and is now Ade9, leaving behind ${\mathrm{PP}}_{i}$. For clarity, we refer to the Ade9 backbone phosphate still as $P_{\alpha }$ as it was on the dATP ligand in the reactant state, and the ${\mathrm{PP}}_{i}$ is the $P_{\beta }$ and $P_{\gamma }$ phosphate moieties. The 3'OH of Thy8 is still coordinated to Mg_A_.

**FIGURE 5:**
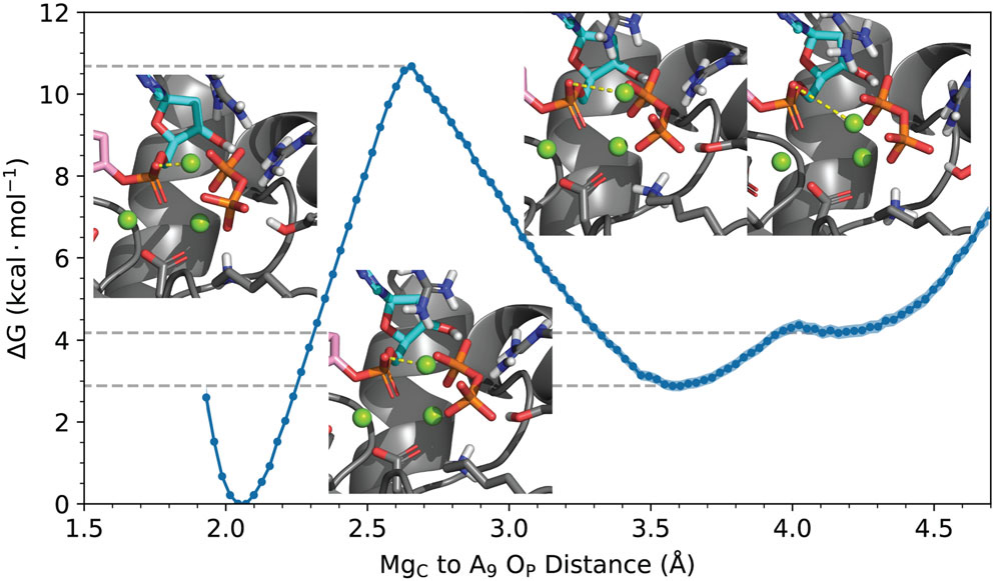
Potential of mean force for the transfer of Mg_C_ (in the product state with the m12‐6‐4 ion parameters) from the backbone phosphate of the growing DNA primer strand's Ade9 to the pyrophosphate only (${\mathrm{PP}}_{i}$). Gray dashed lines help demark the free energy of the transition state and two local minima product states. Snapshots correspond to reactant, transition, and two product states (from left to right). The dashed yellow line in the structures shows the collective variable used for the umbrella sampling.

In the three‐Mg^2+^ simulations, the GAFF2 12‐6 and 12‐6‐4 ion models both lead to Mg_C_ binding to oxygens from each phosphate moiety (α,β,andγ) at the same time (as can be seen in the left‐most structure of Figure 5). The product state has lost a proton and has a formal charge of one less than the reactant state; these oxygens with greater negative partial charges are excellent chelators for Mg_C_. In the m12‐6‐4 simulations, three of the five WT simulations start with Mg_C_ bound to all three phosphate moieties, but the other two only show contact with $P_{\beta }$ and $P_{\gamma }$ of the ${\mathrm{PP}}_{i}$. These latter simulations, and not those with the crystal structure interactions, show additional differences. Mg_A_ moves away from Thy8 3'O and interacts with the Ade9 $P_{\alpha }$ (this shift can be seen between the two top right structures of Figure 5). There is a DNA backbone conformational change of Thy8 and Ade9 that shifts Ade9 away from its starting configuration and into the solvent accessible protein cavity nearby. In the S113A simulations, all five runs start with Mg_C_ bound to all phosphate moieties and end there. Both WT and S113A proteins with two Mg^2+^ in the starting structure show the same features as the m12‐6‐4 three‐Mg^2+^ simulation where Mg_C_ occupies its alternate binding mode interacting only with ${\mathrm{PP}}_{i}$ and not the phosphate of Ade9. This similarity between the states without migrated and post‐migrated Mg_C_ suggests that Mg_C_ is crucial for stabilizing the product state as seen in the crystal structure.

Given the migration of Mg_C_ from $P_{\alpha }$ to ${\mathrm{PP}}_{i}$ observed in the m12‐6‐4 simulations, we ran umbrella sampling with these ion parameters to probe the free energy profile of this process (Figure 5). The optimal coordination distance between the Ade9 phosphate backbone oxygen and Mg_C_ is around 2.08 Å and the transition state occurs with a barrier of approximately 10.6 $\text{kcal}\cdot {\mathrm{mol}}^{-1}$ at a distance of 2.65 Å. The first product well is at a distance of 3.6 Å and is ≈ 3 $\text{kcal}\cdot {\mathrm{mol}}^{-1}$, with the second product well being a barely metastable state (4.2 Å) at just above 4 $\text{kcal}\cdot {\mathrm{mol}}^{-1}$. The migration of Mg_C_ appears to be on pathway to product dissociation. Overall, while the energetic profile for Mg_C_ migration is not very flat, this event was observed in two out of the five unbiased simulations, suggesting that relatively subtle structural rearrangements in the active site may have a notable impact on the most favorable coordination mode of Mg_C_. It is worthwhile to compare this FES with more sophisticated models, such as DFTB3/MM. However, considering the substantial computational cost (since a large number of water molecules need to be included in the QM region to allow a consistent treatment of Mg_C_'s environment during migration), we leave the comparison to future work.

### 2.3 DFTB3/MM calculations generally agree with AMBER simulations

**FIGURE 6:**
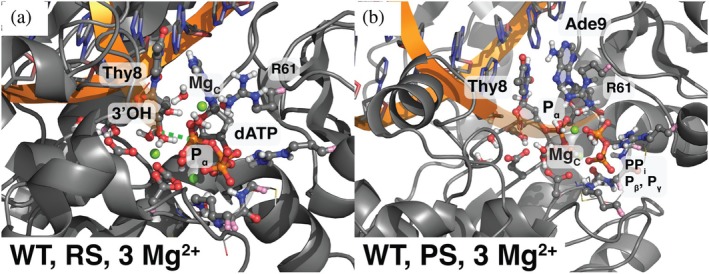
Snapshots of the last frame (of one replicate) of the 555 ps DFTB3/MM GSBP MD simulations for the recant and product states with three Mg. The QM region is shown in ball and stick format (including the visible waters), but all other solvent and side chains are hidden for clarity. The exception is that side chains shown as thin lines (near the right edge) represent the MM region for the continuous QM backbone selection used to ensure the ligand phosphate moieties are not directly interacting with MM atoms. Atoms are colored according to: C = gray, H = white, N = blue, O = red, P = orange, QM Link H Atoms = pink. The 3'O to $P_{\alpha }$ distance in the RS is shown as the dashed green line.

To compare with classical MD simulations with MM force fields, we also conduct DFTB3/MM simulations (setup examples in Figure 6) for the same combinations of proteins, states, and number of Mg^2+^ ions. The DFTB3 parameterization for Mg and Zn has room for improvement in metal–ligand interactions with mean absolute deviations (MAD) of 3–5 $\text{kcal}\cdot {\mathrm{mol}}^{-1}$ but showed excellent geometries with single point energy calculations at a higher level of theory, resulting in MAD of around 1 $\text{kcal}\cdot {\mathrm{mol}}^{-1}$ (Lu et al., 2015). Recent examples of application of these parameterizations to metalloenzymes include alkaline phosphatase (Zn^2+^) (Deng & Cui, 2023; Hou & Cui, 2013; Roston et al., 2016; Roston & Cui, 2016), USB1 (Nomura et al., 2018), myosin (Lu et al., 2017) and DNAP (Roston et al., 2019) (Mg^2+^), which included a benchmark of pK_*a*_s showing good agreement when coordinated to these elements.

For each system, we simulate 555 ps, which represents shorter but a respectable amount of sampling for QM/MM simulations. We use the generalized solvent boundary potential (GSBP) (Im et al., 2001; Schaefer et al., 2005) and thus lose the global flexibility of the complex (see Methods). This helps minimize CHARMM force field's limitations, used here for the MM portion. None of the simulations show disruption to the active site in the reactant state on this timescale, with key distances maintained that match with the AMBER simulations, such as the nucleophilic attack distance of the Thy8 3'O to $P_{\alpha }$ as shown in Figure S.1. Interestingly, all the reactant simulations with three Mg^2+^ show coordination of Mg_C_ to the dATP nitrogenous base nitrogen at a distance of around 2.1 Å (Figure 6), which may be overestimated in strength since the m12‐6‐4 ions are parameterized to get the binding free energy correct for such interactions (Panteva, Giambasu, & York, 2015b). An alternative explanation is that there are not enough QM waters to complete the Mg_C_ coordination shell, since some of the QM water molecules (10 in total, initially selected based on the distance to Mg_A_) dispersed in the active site during the MD simulations.

In the reactant state simulations, Mg_C_ stays coordinated to $P_{\alpha }$ in all runs for both WT and S113A proteins. In one of the S113A runs, Mg_C_ becomes coordinated also to $P_{\gamma }$, which results in the 3'O to $P_{\alpha }$ distance being elongated slightly from 3.2 to 3.4 Å. This rearrangement results in no change to the Mg_A_ distance. In another run of the three‐Mg^2+^ S113A simulations, however, a very severe lengthening of both of these key distances occurs and the interactions are broken. In the S113A simulations with two Mg^2+^, two of the runs show a slight deviation of the distance at 3.4 Å, and one run has a jump in the 3'O to $P_{\alpha }$ distance to above 4.0 but returns without any noticeable distribution to the active site. One of the WT two‐Mg^2+^ simulations also shows a slight increase of this distance to 3.4 Å, but none for the three‐Mg^2+^ case.

In the product state, Mg_C_ always stays bound to the $P_{\alpha }$ backbone of Ade9. The interaction is also retained with the ${\mathrm{PP}}_{i}$ $P_{\beta }$ in all but one run of the WT protein (Figure 6). Simulations show that Mg_C_ can interact with $P_{\gamma }$. This is not the same as the Mg_C_ migration observed in the classical simulation with the AMBER m12‐6‐4 force field. Rather, in three of the WT and two of the S113A simulations the $P_{\gamma }$ fluctuates in the active site in a hinge rotation motion (where the $P_{\beta }$ does not move much) such that it moves close to and then away from Mg_C_, which is stable in coordination with the other two phosphates.

The base‐pairing differences of Thy8 at the end of the primer strand are actually more similar to the results of the classical simulation with CHARMM than those with AMBER (Figure S.2). The fluctuation of the distance covers a broad range with DFTB3, but never reaches the same extreme values as with the CHARMM force field. The fluctuations of the base‐pairing distance are higher in the two‐Mg^2+^ case than with three‐Mg^2+^, perhaps in part due to the dATP base coordination discussed above. The fluctuations of this base‐pairing decrease in the product state.

Additionally, a comparison of the behavior of the R61 residue (near the triphosphate tail of the dATP ligand in the reactant state and ${\mathrm{PP}}_{i}$ in the product state) for the different force fields and DFTB3/MM is included in the Supplementary Materials and shows that DFTB3/MM agrees best with the m12‐6‐4 AMBER simulations.

In conclusion, the DFTB3/MM simulations show the same stable active site structure as the AMBER simulations (3'OH distance to $P_{\alpha }$ and Mg_A_, as well as hydrogen‐bonding patterns), similar behavior of Mg_C_ and Arg61 in both reactant and product states. DFTB3/MM is different from AMBER only in sugar pucker distributions (but this is also different from CHARMM) and one measure of base‐pairing (which is more like CHARMM).

### 2.4 Sugar pucker

From the experimental time‐resolved crystal structures, the sugar pucker of the terminal base (Thy8) is C2'‐endo in the reactant state and C3'‐endo in the product state (Gregory et al., 2021; Nakamura et al., 2012). C2'‐endo pucker is also known as southern puckering and is associated with the most common DNA structure, B‐form DNA, while C3'‐endo (northern) puckering is associated with RNA structures and A‐form DNA (Dickerson et al., 1982; Zgarbová et al., 2018). The CHARMM force field can most correctly reproduce C3'‐endo puckering associated with A‐form DNA and hybrid RNA/DNA duplexes (Knappeová et al., 2024) (even if the A‐form is not stable enough (Jurečka et al., 2024)), and this is not true for the AMBER force fields. The balance between C3'‐endo and C2'‐endo puckering is an ongoing challenge for AMBER nucleic acids, with the A‐form DNA being too unstable compared to B‐form DNA, resulting in the newest OL24 AMBER force field (Jurečka et al., 2024; Zgarbová et al., 2018, 2025). Despite these limitations, we see the correct experimental sugar puckering for the reactant state terminal Thy8 in both AMBER and CHARMM simulations. While the overall puckering is the same, the distributions are different in their shape and width, suggesting they do not share the same dihedral potentials (see Figure S.3), a point of consideration for future force field parameterizations.

In the product state simulations with the m12‐6‐4 ion parameters, we observe the same trends as with the DFTB3/MM simulations. The product sugar pucker is C3'‐endo while Mg_C_ is present. When Mg_C_ is left out of the simulation, or when Mg_C_ migrates away from the Ade9 $P_{\alpha }$, the sugar pucker immediately undergoes a change, which is coupled to a DNA backbone conformational change. This suggests that the product state's unique C3'‐endo configuration is not a result of innate DNA puckering energetics but rather a configuration that Mg_C_ locks into place.

DFTB3/MM simulations show the correct qualitative shift in the product (see Figure S.4), although the sugar puckering profile in our simulations was quite poorly reproduced overall. This is especially noted with the reactant state simulations where the pucker of the crystal structures is at the tail end of DFTB3 puckering distributions. We have thus performed ab initio calculation with MP2 to quantify this (see, e.g., Figure S.6). MP2 has been known to be a reliable method that matches experimental puckering distributions and higher‐level coupled cluster calculations (Foloppe & MacKerell, 1998; Huang et al., 2014; Islam & Roy, 2012; Liebl & Zacharias, 2021, 2023; Mládek et al., 2010, 2014), and we also test several popular DFT functionals for comparison. As discussed more extensively in the Supplementary Materials, the DFTB3 puckering scans on models systems (Figure S.7) show that the energetics of minima and barriers are often reproduced poorly, and ring structures are often too flat, indicating that torsional barriers are too low (see Figures S.8–S.13). DFTB3 may match the product C3'‐endo state partially due to the external forces from Mg_C_ while it matches less well in the other states since the sugar conformation is not locked down. The DFTB3 energy scan surfaces are different from Huang et al. (2014) because we use different model systems and use restraints to avoid intermolecular hydrogen bonding, but both works agree on the limitations of DFTB3 (Huang et al., 2014). The DFT methods all perform reasonably similarly to MP2, with an edge going to the SCAN0 + VV10 or REVSCAN0 + VV10 methods over B3LYP + D3(BJ).

## 3 DISCUSSION

We begin by discussing the limitations of the CHARMM force field that would lead to incorrect mechanistic conclusions. We then discuss the benefits of the AMBER force field with a focus on different ion parameters. Finally, we draw mechanistic conclusions based on the AMBER force field simulations as to the role of Mg_C_, R61, and the S113A mutation. For comments on the ion parameters of the CHARMM force field, please see the Supplementary Materials.

### 3.1 Limitations of the CHARMM force field

Our CHARMM simulations showed significant drift of the dATP ligand in the active site—the ligand moved out of alignment for nucleophilic attack and Mg_A_ moved away from the 3'OH of the growing strand, two key interactions of the reactant state enzyme substrate complex. The S113A mutation had more disruption than the WT, but Mg_C_ ameliorated these issues, albeit not completely. Drift configurations look like mismatched dNTP ligands in the active site, which adopt incorrect positioning due to a lack of binding energy from the base pairing hydrogen bonds with the template strand (Chang et al., 2022). Our simulations thus suggest that the CHARMM force field underestimates the strength of base‐pairing. These caveats of the CHARMM nucleic acid interactions are also manifested with the DNA duplex fraying at the non‐reactive end, which should be a rare event (Zgarbová et al., 2014). Finally, Thy8 at the reactive end of the growing DNA strand has large variations in the hydrogen bond distance with its template Ade5 that reach up to ~7 Å at the highest without the base‐pairing becoming truly “broken.” Several previous studies have noted similar behaviors with the CHARMM force field (Dans et al., 2017; Jurečka et al., 2024; Minhas et al., 2019). The use of extra sHBfix potential terms with CHARMM36 to prevent base‐fraying and flipping (Knappeová et al., 2024; Kührová et al., 2016), despite the benefits, are not routine to include in CHARMM simulations and the stabilizing strength is an empirical factor. Thus the CHARMM force field challenges are best solved through reparameterization of the nucleic acid model in future work.

The Mg_C_ ion helps stabilize the dATP ligand in the unstable CHARMM active site by reducing its conformational flexibility and “locking” it in place. The S113A mutation disrupts the active site through solvent structure in the active site, as shown previously (Gregory et al., 2021). Nothing here invalidates the work of Gregory et al. (2021) since simulations in that work included Mg_C_ in all cases and were focused on the effect of the S113A mutation and the effect of deoxyribose versus ribose of the primer strand's 3'OH terminal base (sugar pucker differences) (Gregory et al., 2021). In fact, recent work has suggested that the CHARMM force field captures correct sugar puckering distributions in nucleic acids (Knappeová et al., 2024). Additionally, both the AMBER and DFTB3/MM simulations agree with CHARMM that a water network in the active site is disrupted by the S113A mutations.

### 3.2 Ion parameters in the AMBER force field

In general, the AMBER OL15 force field provides a reliable representation of DNA. Although the dATP ligand is observed to shift in a few simulations, all occurred without the Mg_C_ bound and more with the S113A mutation. Experimentally, the primer with dT at the reactive end in S113A has defective binding due to the combination of weaker base‐stacking energy and lack of the S113 hydrogen bond to the primer 3'OH (Gregory et al., 2021); the latter was assessed by dNTP $K_{m}$ values for the S113A mutant with all primer terminal bases (Gregory et al., 2021). This experimental observation validates the effect of the S113A mutation in our simulations as causing qualitatively weaker dATP binding. Additionally, half of these six drift simulations occur with the GAFF2 12‐6‐4 force field for the dATP ligand, suggesting that the GAFF2 parameterization is adequate but has slightly weaker base‐pairing than the OL15 nucleic acid force field. Finally, it should be noted that while the template base hydrogen bond between Thy8 and Ade4 is unstable in CHARMM simulations, it is very stable with the AMBER force fields.

Among the AMBER Mg^2+^ models tested here, we find the m12‐6‐4 parameters are the best for the current system. The reasonable explanation for their success is that they were parametrized to correctly reproduce experimental binding free energy to a model phosphate system and were used in similar mechanistic studies (Panteva, Giambasu, & York, 2015b). Other ion parameters overestimate the binding free energy to dimethylphosphate (experimentally −1.43 $\text{kcal}\cdot {\mathrm{mol}}^{-1}$) by 2–8 $\text{kcal}\cdot {\mathrm{mol}}^{-1}$ depending on the parametrization and water model used while the m12‐6‐4 ion parameters are calibrated at −1.39 $\text{kcal}\cdot {\mathrm{mol}}^{-1}$ (the original 12‐6‐4 model predict −4.56 $\text{kcal}\cdot {\mathrm{mol}}^{-1}$) (Panteva, Giambasu, & York, 2015b). Reparameterization also decreases the unbinding barrier of Mg^2+^ from the phosphate: the barrier is 13.5 $\text{kcal}\cdot {\mathrm{mol}}^{-1}$ for 12‐6‐4 ions and 12 $\text{kcal}\cdot {\mathrm{mol}}^{-1}$ for the m12‐6‐4 ions—both are in agreement with the experimental value of 12.7–13.3 $\text{kcal}\cdot {\mathrm{mol}}^{-1}$ (Allnér et al., 2012).

Indeed, there are unexpected behaviors with the GAFF2 and OL15 12‐6‐4 ion models and the GAFF2 12‐6 models that we do not observe with the m12‐6‐4 parameters. The GAFF2 12‐6‐4 model leads to Mg_C_ migration to $P_{\gamma }$, which is not the binding location in the crystal structure as needed for the chemical reaction (Chang et al., 2022; Gao & Yang, 2016; Gregory et al., 2021). Additionally, since Mg_C_ appears required experimentally for catalysis in all cases (Chang et al., 2022; Gao & Yang, 2016; Gregory et al., 2021), we would not expect significant differences in the Mg_C_ binding propensity in S113A and WT proteins, which rules out the GAFF2 12‐6 and 12‐6‐4 models; the OL15 12‐6‐4 ions also show more binding to the S113A mutant than WT. In the product state, all ion parameters lead to strong binding of Mg_C_. Experimentally, no Mg_C_ is seen in the reactant state structures, and its density is present as product formation occurs (Nakamura et al., 2012), except in the case where incorrect dNTP mismatch with a slow chemical step shows Mg_C_ binding before product formation begins (Chang et al., 2022). Knowing that the m12‐6‐4 model treats the binding free energy mostly correct and the simulation trends agree with available experimental observations, it is likely that Mg_C_ does not stay bound in the reactant state with a high population. Weak binding in the reactant state leading to a low bound population would explain limited evidence of Mg_C_ in the reactant state crystal structures. Conversely, strong binding in the product state is visible due to a much larger population. Thus we suggest the use of the m12‐6‐4 model in protein‐nucleic acids systems.

### 3.3 Roles of Mg_C_ and S113

From the m12‐6‐4 and DFTB3/MM simulations, we observe that the presence or absence of Mg_C_ has profound effects on the local electrostatic environment and solvation structure in the active site. With both MM and QM/MM, Arg61 binds to the dATP ligand in the active site only when there are two Mg^2+^ ions, it prefers other alternate conformations when Mg_C_ binds. This is inline with the crystal structures where Arg61 rotates to an alternate conformation when Mg_C_ is bound (Nakamura et al., 2012). In the product state, we observe that Arg61 is more likely to bind to the ${\mathrm{PP}}_{i}$ ligand even when Mg_C_ is bound. This is in line with Arg61 being sufficient in providing electrostatic stabilization in the reactant state for the dATP ligand without Mg_C_. In the product state, the net charge is one less, thus the active site can accommodate both Mg_C_ and Arg61.

Additionally, Mg_C_ is needed in the product state in order to stabilize the conformation of the DNA (backbone chain and Ade9) found in the product state crystal structure. The shifted configurations do not have the same Thy8 sugar puckering as seen in the product state crystal structure (C3'‐endo), while the simulations with Mg_C_ bound do. This adds evidence for the transient and weak Mg_C_ binding in the reactant state and strong binding in the product state. If Mg_C_ were to bind exclusively to the product state, our simulations would suggest that, prior to Mg_C_ binding, there would be an altered DNA conformation and a different sugar pucker of the nucleophilic primer base. This can be tested experimentally by capturing a crystal structure of DNAP with a DNA duplex and high concentration of pyrophosphate with differing stoichiometric amounts of Mg^2+^ to populate or leave free the third binding site.

The DFTB3/MM simulations show the same effect of the S113A mutation as the MM simulations, which show that the S113A mutation is likely to cause active site disruption more than the WT. We have already noted the exaggerated effects due to the weak base‐pairing and too strong Mg^2+^ binding with CHARMM36. From the m12‐6‐4 AMBER simulations, we postulate that the role of S113 is to be a hydrogen bond acceptor or simply interact with the ordered hydrogen bonding network in the reactant state. From the dATP unbinding simulations, we see S113 interacts directly as a donor to the Thy8 3'OH once the reactant ternary state is no longer formed. This shows why the S113A mutant affects template primer docking, even when the direct interaction was only seen with CHARMM and not AMBER in the reactant state (Gregory et al., 2021). This also aligns with the ground state crystal structure from Nakamura et al. (2012), which shows before Mg_A_ binds, S113 is in position to hydrogen bond with the primer terminal 3'O thymine (even through hydrogen atoms are not visible to inform which is the hydrogen‐bond donor) (Nakamura et al., 2012).

## 4 CONCLUSION

In this work, we highlight the strengths and weaknesses of different simulation methodologies and force field parameters in the context of a biologically relevant metalloenzyme, DNA polymerase η. Our initial simulations with the CHARMM36 force field showed an unstable catalytic center due to the weak base‐pairing interactions between the incoming dATP ligand and the template strand, and weak base‐pairing between the growing and template DNA strands leading to fraying events on the non‐reactive end. Due to the limitations of these simulations, direct interpretation would have led to incorrect conclusions about the mechanistic roles of the third Mg^2+^ ion and the S113A mutation. Additionally, the Mg^2+^ parameters of CHARMM36 are likely also not balanced for phosphate binding, which is of benefit for “artificial” stabilization of the active site, but more balanced parameters should be used for both ions and nucleic acids.

Instead, we find the AMBER OL15 force field to be a better description of DNA properties, as noted in other works. The specific Mg^2+^ ion model used for these simulations also shows critical importance for correctly interpreting the role of Mg_C_. In addition to experimental results, we use DFTB3/MM simulations as a reference to compare the effects of simulation force field parameters. Based on the comparisons with experimental evidence and DFTB3 QM/MM simulations, we conclude that the m12‐6‐4 Mg^2+^ parameters, with their careful parametrization for similar mechanistic studies, are crucial to capture the correct qualitative behavior in Mg_C_ binding in both the product and reactant states, the Arg61 conformational differences thereof, and the role of the S113A mutation.

Taken all together, the simulations suggest that Mg_C_ binds weakly in the reactant state and strongly in the product state, with important effects on the R61 conformation and in stabilizing the product state, as seen in the crystal structures. S113 is most critical as an anchor to the primer before the dATP ligand binds to form the enzyme‐substrate complex and in the reactant state as a hydrogen bond acceptor to the growing strand's terminal 3' OH (occasionally directly but usually mediated through a water molecule). Along with the DFTB3/MM simulations, the classical simulations find that the S113A mutant disrupts the hydrogen bonding water network of the active site.

We also note the shortcoming of DFTB3 for sugar puckering through QM/MM simulations that do not correctly capture experimental sugar puckering values and through comparison with DFT and MP2 potential energy scans for model compounds. Overall, our results show that less than ideal force fields or semi‐empirical QM methods can skew the interpretation of mechanistic issues. Thus, it is important to carefully analyze simulation results and check their consistency among experimental results, multiple force fields, and higher level methods whenever possible. Along this line, it is also of major interest to explore polarizable force fields (Jing et al., 2019; Lemkul et al., 2016) for protein‐DNA systems, since interactions between divalent ions and highly charged groups, such as phosphates, are likely better treated with polarizable models (Baral et al., 2024; Puyo‐Fourtine et al., 2022). On the other hand, as also highlighted by recent studies (Liu et al., 2025; Prusty et al., 2025), it remains important to calibrate these advanced models for a balanced treatment of bonded and non‐bonded terms for structure and dynamics.

## 5 MATERIALS AND METHODS

DNAP in the reactant state (both WT and S113A) was setup from PDB 5KFG (Gao & Yang, 2016) and solvated in a 0.15 M NaCl solution with cubic periodic boundary conditions (105 Å length) with CHARMM‐GUI PDB Reader & Manipulator and Solution Builder (Jo et al., 2008, 2014; Lee et al., 2016, 2020; Park et al., 2023). To explore the effect of the third magnesium in the C‐site, a Mg^2+^ was placed manually near the α‐phosphate to represent the Mg_C_ binding site found in previous work (Gregory et al., 2021). In total, there were 4 systems representing all combinations of two versus three Mg^2+^ ions, and WT versus S133A mutant protein. Each of these systems was simulated with two different force fields. In one set of simulations, the CHARMM36m force field (MacKerell et al., 1998, 2004; Vanommeslaeghe et al., 2010) was used for all components (dATP was modified from the ribose ATP (Gregory et al., 2021)). Additionally, AMBER force fields used ff19SB for proteins (Tian et al., 2020), OL15 for DNA (Galindo‐Murillo et al., 2016), GAFF2 (Wang et al., 2004) for the ligands, the monovalent ion parameters of Joung and Cheatham III (2008), and the divalent Li & Merz ion parameters (the 12‐6 normal usage set) (Li et al., 2013, 2020). In both cases, water was described with the TIP3P water model (Jorgensen et al., 1983).

For each system, four independent MD simulations were run with OpenMM 7.7 (Eastman et al., 2017). Each run underwent 5000 steps of minimization, 125 ps of NVT equilibration at 1 fs time steps at a temperature of 303.15 K using Langevin dynamics (friction coefficient of 1 ${\mathrm{ps}}^{-1}$). Particle Mesh Ewald (PME) was used to describe electrostatics with an error tolerance of $5\times 10^{-4}$ (Darden et al., 1993; Essmann et al., 1995). The non‐bonded cutoff was set to 9 Å. During heating, harmonic position restraints were placed on protein and nucleic acid backbone atoms (400 $\mathrm{kJ}\cdot {\mathrm{mol}}^{-1}\cdot {\mathrm{nm}}^{-2}$) and side‐chain (equivalently the nucleic acid base) atoms (40 $\mathrm{kJ}\cdot {\mathrm{mol}}^{-1}\cdot {\mathrm{nm}}^{-2}$). The OpenMM simulations with 12‐6 force fields here also had a harmonic restraint on the dATP ligand (400 $\mathrm{kJ}\cdot {\mathrm{mol}}^{-1}\cdot {\mathrm{nm}}^{-2}$). No restraints were included on Mg ions. This is acceptable for the Mg_A_ and Mg_B_ ions as they are coordinated by restrained protein residues. Key equilibration time series involving Mg ions are shown for all classical simulations performed in this work in the online code repository, with select graphs for Mg_A_ and Mg_C_ also reproduced in the Supplementary Materials. The traces of all Mg_A_ metrics show normal fluctuations, confirming that the simulations are not artificially trapped and thus the lack of restraints are unlikely to contribute to alternate interpretations of downstream events (Figures S.14 and S.15 in the reactant state; Figures S.17 and S.18 in the product state). Simulations with Mg_C_ leave the ion free to adjust during minimization and equilibration, and some runs do show disruption of the initial placement (Figure S.16 for the reactant state; Figures S.19–S.21 in the product state). However, as the starting coordinates are identical between force fields this is a robust way to identify systematic differences between them. As the starting position of Mg_C_ ion is at the same relative location in the product state setup, this is also a robust way to identify systematic differences between reactant and product states.

Then a 110 ns production run was undertaken in the NPT ensemble using a time step of 2 fs and the Monte Carlo barostat for isotropic pressure coupling at a pressure of 1 bar (Åqvist et al., 2004). The SHAKE algorithm was used to constrain bonds containing hydrogen (Ryckaert et al., 1977). Coordinates were written every 5 ps during heating, and every 100 ps (0.1 ns) during production runs.

The DNAP product state bound to pyrophosphate ${\mathrm{PP}}_{i}$ was setup from PDB 5KFH (Gao & Yang, 2016). The starting place of Mg_C_ was the crystal structure location, bound near the phosphate oxygen atoms of both $P_{\alpha }$ and $P_{\beta }$. The Amber20 PMEMD GPU executable (Case et al., 2020; Gotz et al., 2012; Le Grand et al., 2013; Salomon‐Ferrer et al., 2013) was used to run simulations with the above Amber force fields but various 12‐6‐4 style ions and the TIP4P‐Ew model (Horn et al., 2004, 2005) for both the reactant and product states of DNAP. All of the previously mentioned simulation details were used unless specified. Simulations used the long range dispersion correction included by default (Shirts et al., 2007). Bonds involving hydrogen (not on water) were constrained with the SHAKE algorithm (default tolerance of $10^{-5}$), while waters were kept rigid with the SETTLE algorithm (Miyamoto & Kollman, 1992). Electrostatics were described by PME with the default parameters of the Amber PMEMD engine.

Three sets of simulations with different matching of the 12‐6‐4 ion parameters were used to describe the Mg^2+^ ions. For the dATP ligand, these are the GAFF2 force field with 12‐6‐4 ions (Li et al., 2015a, 2015b; Li & Merz Jr, 2014), the same ions but the OL15 force field applied to the nucleotide parameters, or the Panteva, Giambasu, & York (2015b) m12‐6‐4 ion parameters with the OL15 parameters for the nucleotide (Panteva, Giambasu, & York, 2015b). In the cases of OL15 force fields and thus also the m12‐6‐4 ion case, the nucleoside region was described completely this way and the triphosphate tail used bond/angle/dihedral parameters from Meagher et al. (2003) but with the all non‐bridging phosphate oxygens of the tail using the phosphate backbone oxygen LJ parameters of the DNA OL15 force field (Meagher et al., 2003). For the ${\mathrm{PP}}_{i}$ ligand, bond/angle/dihedral terms were always described with the GAFF2 force field but all non‐bridging phosphate oxygens had again the LJ parameters of the OL15 force field when used in the OL15 12‐6‐4 and m12‐6‐4 simulations. The bridging phosphate oxygens of the dATP ligand have their atom types changed from the original GAFF2 parameterization to match the atom types from the DNA OL15 force field. However, in the case of the original Panteva, Giambasu, & York (2015b) m12‐6‐4 corrections, the LJ correction is applied only to the non‐bridging oxygen atom type of phosphate, so treatment is consistent when comparing the OL15 12‐6‐4 and the m12‐6‐4 simulations. The bridging phosphate oxygens of the pyrophosphate are treated with the GAFF2 parameters in both GAFF2 and m12‐6‐4 simulations (we did not perform OL15 12‐6‐4 simulations for the product state) and thus are consistent when comparing the two force fields. For each of ion parameter sets, 5 independent MD simulations were run exactly as described above, except for a total production simulation length of 330 ns.

DFTB3/MM simulations using the GSBP followed previously reported protocols from Roston et al. (2019) (see Supplementary Materials). Methods for the umbrella sampling calculations and sugar puckering DFT benchmarking can be found in the Supplementary Materials.

Simulations were visualized by VMD (Stone, 1998; Frishman & Argos, 1995; Humphrey et al., 1996) and PyMOL (Schrödinger, LLC, 2025). Trajectory statistics and analysis were calculated with MDAnalysis (Gowers et al., 2016; Michaud‐Agrawal et al., 2011; Smith et al., 2019). We used the five‐membered sugar puckering pseudo‐rotation angle definition of Cremer and Pople (1975) as implemented in MDAnalysis.

## AUTHOR CONTRIBUTIONS

**Reilly Osadchey:** Investigation; formal analysis; writing – original draft; writing – review and editing. **Qiang Cui:** Conceptualization; supervision; funding acquisition; project administration; resources; writing – review and editing.

## CONFLICT OF INTEREST STATEMENT

R.O. and Q.C. have no conflicts of interest to disclose.

## Supporting information


**Data S1:** Supporting Information

## Data Availability

Input coordinates and topologies and non‐third party scripts needed to reproduce the results of this work are openly available in a GitHub repository *ff_matter_dna_pol_eta* at https://github.com/rosadche/ff_matter_dna_pol_eta. Also included are the key distances during equilibration. Other data are available from the corresponding author upon reasonable request.
