# Supplementary material for: Force fields matter in DNA pol η mechanistic analysis
Source: Protein Sci. 2025 Dec 23;35(1):e70348. doi: 10.1002/pro.70348 (PMC12723750; doi:10.1002/pro.70348)
Supplement: Supplementary file 1 — Data S1: Supporting Information [file PRO-35-e70348-s001.pdf]

Supplementary Materials: Force Fields Matter in DNA Pol η Mechanistic Analysis

Reilly Osadchey[†] and Qiang Cui[*,†,‡,¶]

†Department of Chemistry, Boston University, Boston, MA 02215

‡Department of Physics, Boston University, Boston, MA 02215

¶Department of Biomedical Engineering, Boston University, Boston, MA 02215

E-mail: qiangcui@bu.edu

Supplementary Methods

Ligand Unbinding

Umbrella sampling was performed with the Amber22 PMEMD engine and NFE module[1] with all the same simulation details as the main text, except only using the m12-6-4 ion parameters. All simulations used equally spaced harmonic restraints, and each successive window was initiated from the last configuration of the previous window. Collective variables (CVs) had their values written every 500 steps (1 ps). Free energy surfaces were constructed by re-weighting using the Multi-state Bennet Acceptance Ratio (MBAR).[2–4]

For the migration of Mg_C in the product state, from its initial position coordinated to the DNA backbone to only being coordinated to the PP_i, one independent run was performed. This simulation was restarted from the end of a randomly selected molecular dynamics simulation run that had Mg_C still coordinated to the DNA backbone. The reaction was described using the distance of Mg_C from the coordinating DNA phosphate oxygen as the

CV. There were 20 equally spaced umbrella windows (250 $kcal \cdot mol^{-1} \cdot Å^{-2}$) ranging from 2.0 to 4.6 Å. An additional window was added at 2.61 Å with a stronger force constant (400 $kcal \cdot mol^{-1} \cdot Å^{-2}$) to better sample the transition state region. These simulations were re-weighted using every CV value, with no discarded equilibration period.

The unbinding of the dATP ligand alone was originally described using the distance CV between the center of mass (COM) between all ligand atoms and the COM of the C_α of residues Asp16, Met17, Asp118, and Glu119. This is related but not identical to the CV definition in Reference.[5] We used 110 umbrella windows centered equally from 8 to 24.31 Å. For all other unbinding simulations (dNTP with Mg_B and all PP_i simulations), we adjusted the CV to be the distance between ligand COM to the COM of the C_α of residues Asp16, Met17, Ser116, Ile117, Asp118, and Glu119. For the dATP ligand, this was sampled for distances between 7.5 to 24.36 Å over 110 umbrella windows. dATP simulations were restarted from the coordinates (and new velocities) from the end of one WT, two-Mg^{2+} simulation replicate. PP_i simulations were restarted from the coordinates in the product state of previous umbrella sampling simulation where Mg_C has become bound only to PP_i. In all cases, five non-equilibrium pulling simulations were performed where the first window was run for 4 ns to allow time for velocity equilibration, while all other windows were run for 2 ns. Only two of these non-equilibrium runs were selected for final equilibrium umbrella sampling. These were selected as the runs that did not disrupt structure, had a reasonable initial binding profile, and the runs had different number of Mg^{2+} ions coordinating to the ligand. These simulations were re-weighted with CV values sub-sampled every tenth value. Based on re-weighting consecutive 5 ns blocks, the first 45 ns were discarded as an equilibration period.

DFTB3/MM Simulations

The simulation setup followed protocols found in Roston *et al.* (2019).[6] The QM region contained the dNTP ligand; two or three Mg^{2+} ions; 10 waters; side chains (DIV-style link

atoms between C_α and C_β) of Asp13, Arg55, Arg61, S113 (or the S113A mutant), Asp115, Glu116, and Lys231; the backbone between Met14 and Cys16 (side chains excluded); and the first DNA nucleotide of the growing strand. This encompassed around 200 atoms but varied slightly under different simulation setups. The QM region in the reactant state and two Mg^{2+} had a net charge of zero. The region was treated at the DFTB3-D3 level of theory with corrected P parameters for phosphate hydrolysis reactions.[7–13] All MM atoms were treated with the CHARMM36 force field[14,15] with TIP3P water.[16] The QM waters were kept in the active site by use of a Flexible Inner Region Ensemble Separator (FIRES) restraint[17] (centered on the Mg_A atom, with a force constant of 100 $kcal \cdot mol^{-1} Å^{-2}$).

Simulations were setup from the above crystal structures and run with the CHARMM program (Chemistry at HARvard Macromolecular Mechanics).[18–20] Electrostatics were treated with the generalized solvent boundary potential (GSBP) setup which uses implicit solvent to describe the electrostatics from the frozen outer region (solvent dielectric of 80.0 and a protein dielectric of 1.0, up to 20th order spherical harmonic basis functions to describe the inner region.) The mobile inner region was defined as within 27 Å from the Mg_A, and treated with Newtonian dynamics except for a buffer region between the shell between radius 23 Å and 27 Å, which was treated with Langevin dynamics. This buffer region also had scaled harmonic restraints with force constants calculated to reflect the crystallographic B-factors associated with protein heavy atoms.

Five replicate simulations were run for each setup. As above, we tested all combinations of two versus three Mg^{2+}, the WT or S113A mutant, and the reactant and product states. Simulation started with three rounds of minimization using steepest descent: 500 steps with non-frozen waters (exit gradient tolerance of 0.1 $kcal \cdot mol^{-1} \cdot Å^{-1}$), 500 steps for all QM atoms (gradient tolerance of 0.05 $kcal \cdot mol^{-1} \cdot Å^{-1}$), and all non-frozen atoms (gradient tolerance of 0.05 $kcal \cdot mol^{-1} \cdot Å^{-1}$). Equilibration occurred over 150 ps at 1 fs time steps, heating the system from 50 K by 10 K increase every 1 ps until the final temperature of 300 K was reached. The SHAKE algorithm was used to constrain bonds containing hydrogen.[21]

This took 100 hours on 1 central processing unit (CPU). Production simulations at 300 K were run for 555 ps, representing approximately 15 days of CPU time. Coordinates were saved every 100 frames, representing every 0.1 ps.

QM Sugar Puckering Benchmark

Potential of several small model systems for sugar puckering (both the ribose and deoxyribose forms and deprotonation the C2' alcohol) were performed at the DFTB3+D3 level using the CHARMM program (see Figures S.6 and S.7 for the model systems). Initial files for these models (neutral state) were obtained from CHARMM-GUI's ligand reader and modeler.[22] Custom restraints were implemented in the CHARMM program to allow the harmonic restraint of the Z_x, Z_y sugar puckering collective variables for five-membered rings (definition from References[23,24]). Scans ran over all combinations of the Z_x, Z_y values between -60 to 60, with spacing of 6° using a harmonic restraint with a force constant of 10 $\text{kcal} \cdot \text{mol}^{-1} \cdot \text{deg.}^{-2}$. Flat bottom harmonic restraints (force constant of 50 $\text{kcal} \cdot \text{mol}^{-1} \cdot \text{rad.}^{-2}$) were placed on the following dihedral angles when present: on the one (deoxyribose) or two (ribose) alcohol(s) such that the oxygen hydrogen was in plane with the carbon and hydrogen it is attached to $(180 \pm 20°)$, the nitrogenous base such that it could not flip around to form any hydrogen bonding interaction $(270 \pm 60°)$, and on all the dihedral chain connecting the C5' carbon backbone through the methyl-ether of the phosphate group $(180 \pm 20°)$. These were included to prevent any intermolecular interactions between species, which could potentially obfuscate innate puckering energy differences with energy differences for intermolecular interactions. It should be noted that intermolecular contacts do still occur, but are fewer than without restraints and do not form hydrogen bonds. These restraints are justified because without restraints we did see the nitrogenous base rotate around to form hydrogen bonding interactions, and the deprotonated ribose model systems were always "locked" into one stable conformation due to the proton being shared directly between the two alcohol groups. Each configuration was minimized for 2000 steps using the steepest descent algorithm, followed

by 5000 steps using the Adopted Basis Newton-Raphson algorithm (until tolerance on the gradient reached 0.01 $kcal \cdot mol^{-1} \cdot Å^{-1}$). All hydrogen atoms were constrained with SHAKE.

All density functional calculation (DFT) single point energy calculations were performed using the CHARMM interface to the FermiONs++ electronic structure program.[25,26] Its hybrid integral engine can run on Graphics Processing Units (GPUs) and Central Processing Units (CPUs) so we ran each window with 1 GPU and 6 CPUs.[27] By default, FermiONs++ operates exchange in a single precision mode with minimal precision calculations.[28] Additionally, we used the default linear scaling semi-numerical exact exchange,[25,26,29] and integral screening.[30] We used the default grid (Turbomole-inspired gm3 grid, with the final energy evaluated on the gm5 grid upon convergence).[31,32] Electronic convergence was defined as less than or equal to a 10^{-8} atomic unit change on the energy and a 10^{-6} a.u. on the root mean square difference of the density. For DFT methods, we used only hybrid functionals: B3LYP-D3(BJ),[12,13,33–36] the SCAN0 functional[37] with Vydrov-Van Voorhis 10 (VV10) dispersion[38,39] (a non-local correlation energy functional), and the revised SCAN0 (REVSCAN0)+VV10 dispersion.[40]

Given the evidence that Møller–Plesset perturbation theory to the second order (MP2)[41] is a good method to compare sugar puckering given its correlation with experimental values,[23,42] we also perform MP2 calculation with the CHARMM interface to Gaussian 16.C.01[43] using 28 CPUs. All other parameters were the Gaussian program's defaults. All calculations used the 6-311+g(d,p) basis set.

Additional Results and Discussion

Main Text Extended Content

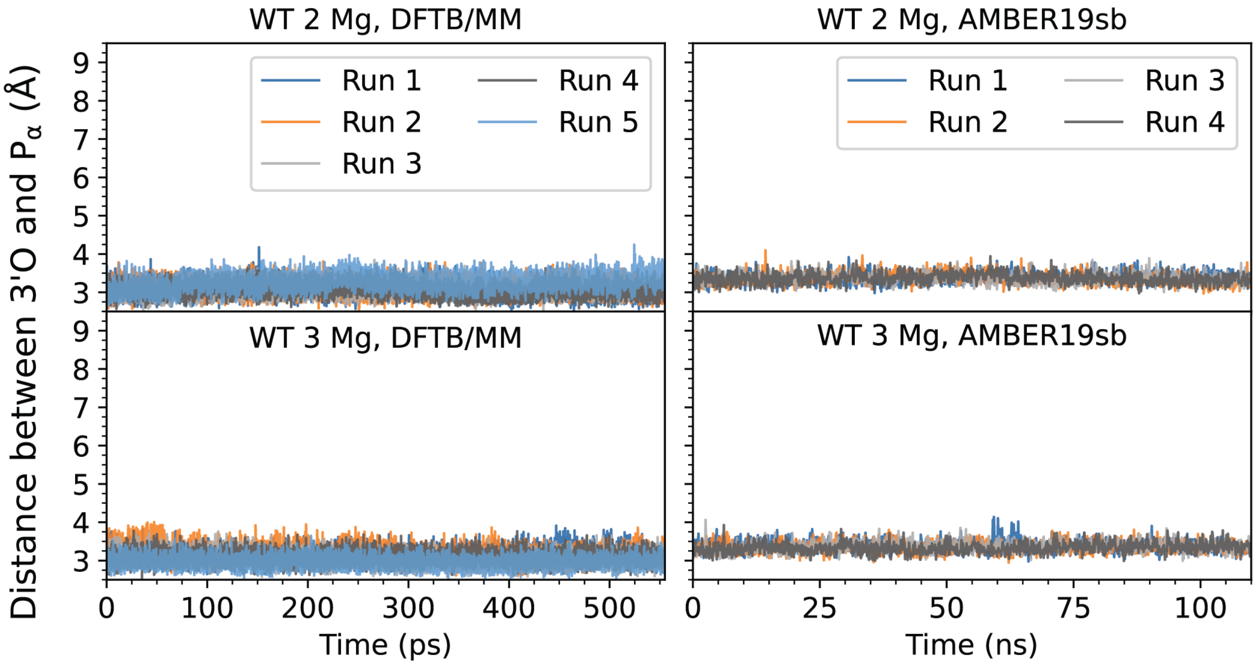

Figure S.1: Time evolution of the nucleophilic 3'O to P_{α} distance for the DFTB3/MM and classical AMBER force field simulations, meant as a comparison to Figure 2 in the main text. All runs show a stable active site.

Table S.1: Simulations with the different AMBER force fields show a small number of replicates with disruption of the catalytic center by the reactant nucleotide dATP drifting away from the nucleophilic 3'OH (as compared to the CHARMM force field). This is only seen with the 12-6-4 ion models, but at a very infrequent rate overall.

Force Field	Protein	Mg^{2+}	Drift (No./5)
GAFF2, 12-6-4	S113A	2	1
GAFF2, 12-6-4	S113A	3	2
OL15, 12-6-4	WT	3	1
m12-6-4	S113A	2	1
m12-6-4	WT	3	1
All other AMBER			0

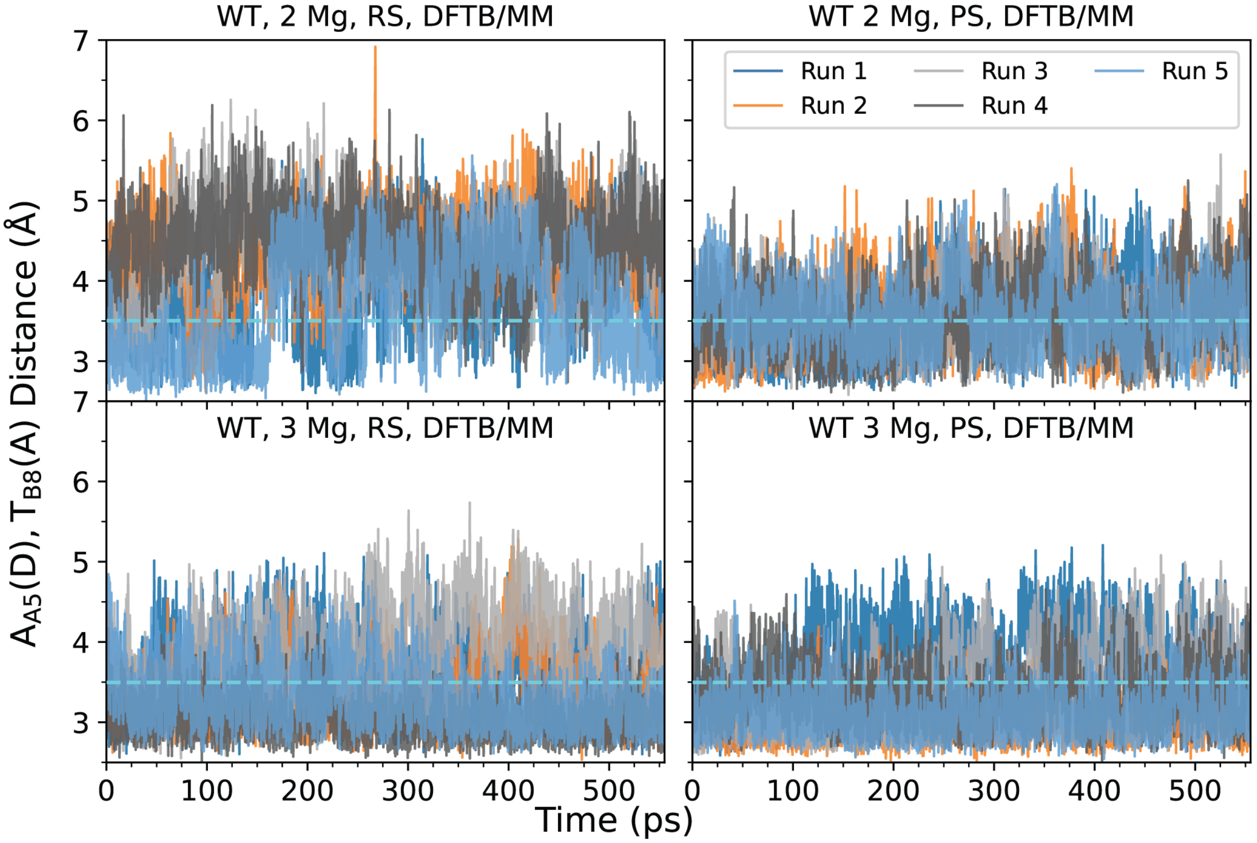

Figure S.2: Time evolution of the base-pair hydrogen bonding of active site terminal base-paired DNA residues in both the reactant and product states for the DFTB3/MM simulations. This graph is the companion graph of Figure 3 (the adenine and thymine residues are colored in yellow in that figure). The blue line in bottom graphs represents the hydrogen bonding donor-acceptor distance cutoff of 3.5 Å.

Active Site Hydrogen Bonding and Sugar Puckering comparison between AMBER, CHARMM, and DFTB3

In the simulations, the S113A mutation removes the S113 hydroxyl as a hydrogen bonding partner which is known to have an effect on anchoring the DNA strand correctly.[44] We defined hydrogen bonds (HB) as a donor-acceptor distance of less than or equal to 3.5 Å, and a donor-hydrogen-acceptor angle of greater than or equal to 150°. This decision was made as the stricter cutoff of 3 Å resulted in very few registered hydrogen bonds with the AMBER force field, making comparison difficult.

In the CHARMM simulations of the WT protein, S113 acts as a HB donor to the terminal Thy8 O3' hydroxyl, the O4' of the Thy sugar ring, to a nearby glutamate E116, or water. The relative frequency of these hydrogen bonding patterns is variable between runs and conditions. In the two-Mg^{2+} simulations, the WT protein predominantly donates a HB to water. The second most common is with Thy8 O3' as a HB acceptor, followed by Thy O4'. The Thy8 O3' as a HB donor interacts with the O4' and O5' of the dATP with similar frequency and occasionally with water. Only in a few frames does S113 act as a HB acceptor from the Thy O3'. In the absence of S113 in the S113A mutant, the Thy8 O3' alters its hydrogen bonding such that it donates predominantly to water. When all three Mg^{2+} are present, the WT simulations show the same HB trend as with two Mg^{2+}. On the other hand, the S113A mutant shows that the Thy8 O3' is a HB donor to the dATP's O5' and O4' atoms, with some but much less HB donation to water.

Given the three-Mg^{2+} case is the state with the most stable active site, this is likely to best represent the true hydrogen bonding pattern of the protein. In this case, S113 donates a hydrogen bond to the Thy8 3'OH in 40 to 90% of the frames of different simulation runs, otherwise it hydrogen bonds to water (10 to 40%). Thy8 donates a hydrogen bond to S113 for about 40 % in one simulation run, but usually hydrogen bonds to the incoming dNTP's sugar O5' (and never to water).

In the AMBER simulations, the S113 hydrogen bonds predominantly to water with very

rare fluctuations that lead to hydrogen bonding to the nearby negatively charged residues of the DED motif. Thy8 donates a hydrogen bond to S113 25 to 40 % of the time, with most of the remaining snapshots with hydrogen bonds donating to water. In the absence of S113, Thy8 hydrogen bonds with water almost exclusively. This trend is not altered by any of the other AMBER ions tested and described in the next section. The only notable difference is that on rare occasions, Thy8 hydrogen bonds to the sugar O5' of the dNTP ligand.

There are hydrogen bond differences between simulations with two and three Mg^{2+} ions in the product state (m12-6-4 ions). The newly added nucleotide, Ade9, exclusively donates a hydrogen bond from its 3'OH to the PP_i. In the m12-6-4 runs with two Mg^{2+} ions, the hydrogen bond can be to any of the non-bridging oxygens of P_β suggesting a lower rotational barrier—the three-Mg^{2+} case shows a single persistent hydrogen bond acceptor when Mg_C is bound. In the simulations with the retained starting configuration of Mg_C, S113 predominantly hydrogen bonds to water with some interaction with E116. In the alternate Mg_C location, S113 hydrogen bonds to E116 or the phosphate backbone oxygen of Ade9. Similar patterning is seen in the two-Mg^{2+} simulations as well. The most hydrogen bonds are donated to the Ade9 backbone, with a similar amount being donated to E116 and the Thy8 3'O (once nucleophile, now phosphate backbone).

The sugar puckering distributions between the CHARMM and AMBER force fields show very different distributions in center and spread, without qualitatively changing the sugar puckering state conclusions (Figure S.3). In the CHARMM three-Mg^{2+} simulations, our stable CHARMM reference, we see the pucker pseudo-rotation angle distributions are peaked around 170°. One simulation starts around 80° but eventually converges to the other main location. These distributions are fairly narrow. In contrast, the AMBER simulations show a wide and gently sloped distribution ranging from (including small probability edges of the distributions) 60 to 240°. Based on common definition, the C2'-endo sugar pucker is defined as a pseudo-rotation angle of 162° and C3'-endo is defined as 18°.[23,45] However, commonly the nearby puckering states are grouped together—one such definition is from Knappeová *et al.*

(2024) where the C2'-endo is defined as the range of values between 135 to 180° and C3'-endo is defined as −10 to 40°.[46] This means the sugar puckering in both cases can be classified as the correct the C2'-endo puckering expected of the reactant state.[47] The differences in distribution shapes suggest the free energy for sugar puckering may be very different in these two force fields, although we have not calculated it here. Despite the distribution differences, crystal structure pucker values could be drawn from either distribution as is illustrated graphically in Figure S.3.

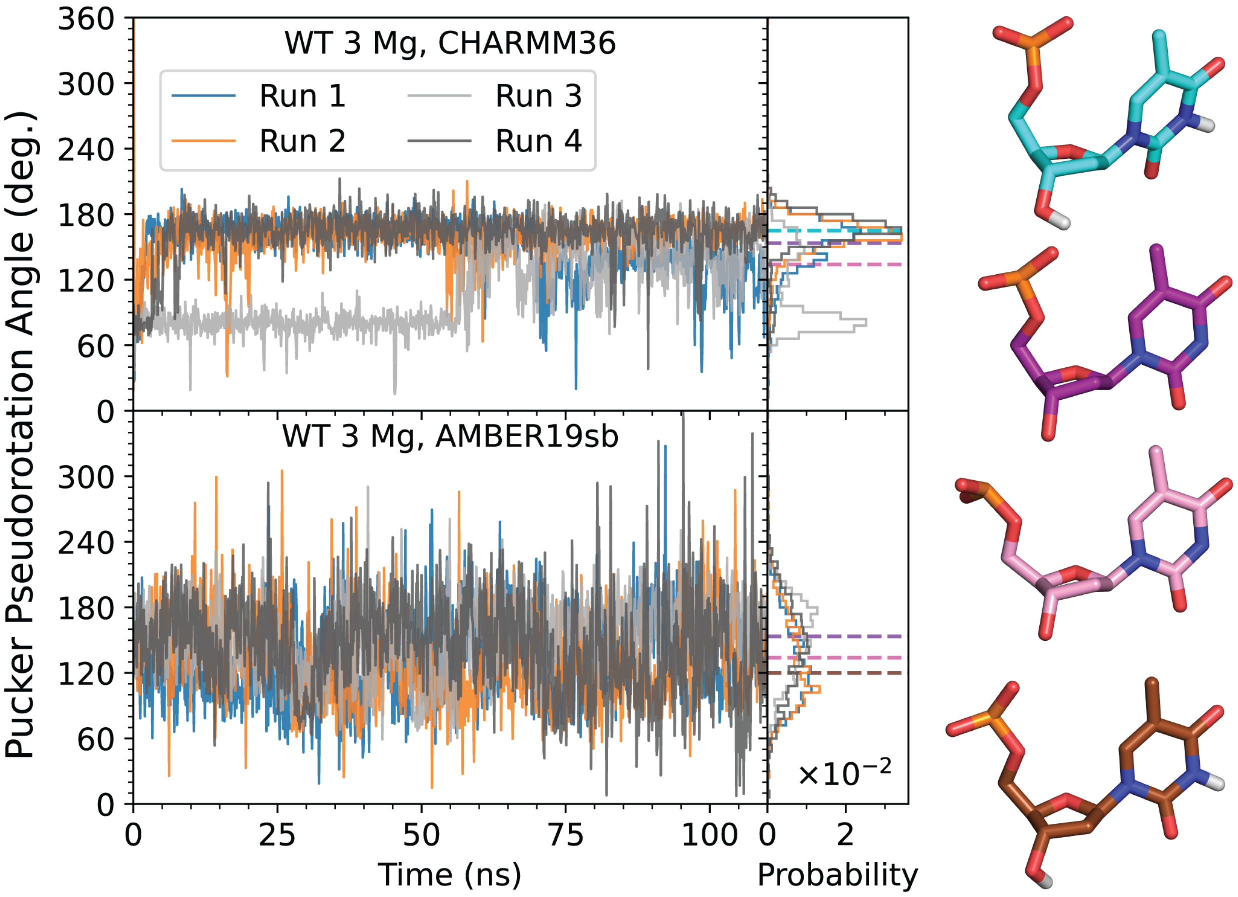

Figure S.3: Sugar puckering distributions of the reactive Thy8 terminal DNA base differ between CHARMM (top) and AMBER force fields (bottom). Example structures are have their location marked on the graph by colored dashed lines. Cyan: representative CHARMM structure, Purple: 5KFH (reactant alternate location), Pink: 5KFG crystal structure, Brown: representative AMBER structure. Atoms are colored according to: C=variable, H=white, N=blue, O=red, P=orange.

Sugar puckering is very different with DFTB3 (Figure S.4) than any of the other force

fields. As discussed in the next section, the energetics of sugar puckering is a major (and known) limitation of DFTB3. In the WT two-Mg^{2+} reactant simulations, the puckering is well defined in the C3'-endo region, which is not experimentally correct. The three-Mg^{2+} reactant state is best defined as O4'-endo and not correct either, although the very edge of the distribution samples the crystal structure puckering values rarely. In the product state, the three-Mg^{2+} simulations are spot on at sampling the correct C3'-endo configurations. This is less true of the two-Mg^{2+} case, which samples a distribution ranging from some of the C3'-endo and O4-endo states. Once again, the presence of Mg_C and its interactions with the P_α backbone of Ade9 prevent DNA backbone and thus sugar pucker rearrangement.

Hydrogen bonding patterns are significantly different among the DFTB3/MM simulation cases, but align well with the conclusions of the AMBER force felids. In the WT two-Mg^{2+} simulations, S113 hydrogen bonds to E116 in two runs, and water in the other three. Thy8 3'OH hydrogen bonds to S113 in a small part of three runs, and to water the rest of the time. In the three-Mg^{2+} case, S113 only hydrogen bonds to E116 in a part of one run (16%), and to water in the rest. Thy8 hydrogen bonds to water almost exclusively. In the product state two-Mg^{2+} simulations, S113 hydrogen bonds to water and in the three-Mg^{2+} simulations to E116 (except in one run to water). In both cases, the hydrogen bonding of the Ade9 3'O is to the PP_i. These trends can be visualized partly in Figure 6. The reactant (left) portion shows a bridging water between Thy8 3'OH and S113. In the product portion (right) of the figure, S113 is hydrogen bonded to E116.

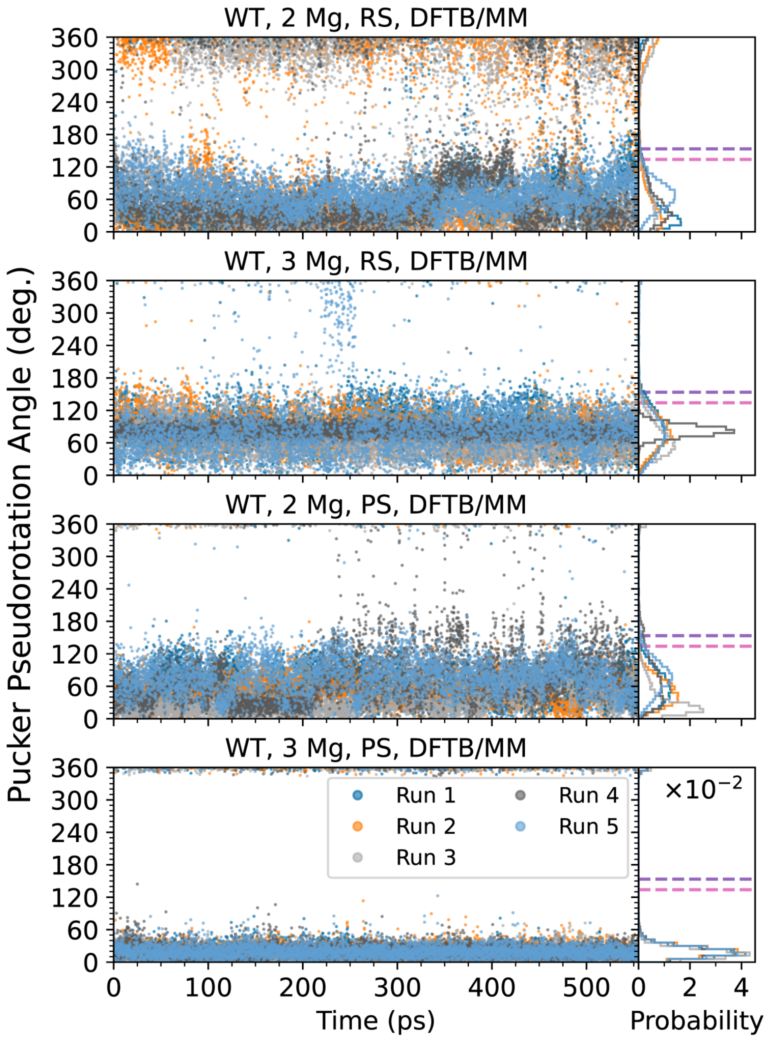

Figure S.4: Sugar puckering time evolution and probability distributions for the DFTB3/MM simulations in the product and reactant states (companion to Figure S.3). The dashed line represent experimental values: Purple: 5KFH (reactant alternate location), pink: 5KFG crystal structure.

Comparison of R61 Configurations in Reactant and Product States

The Arg61 residue near the dATP triphosphate tail or PP_i phosphates also shows different behaviors with different ion force field parameters. In the reactant state, the CHARMM and AMBER 12-6 ion models lead to the same trend. In both the WT and S113A proteins, Arg61 does not stay bound to the phosphates of the dATP ligand. Arg61 has multiple side chain conformations and samples states closer to the ligand phosphates in the two-Mg^{2+} simulations than the three-Mg^{2+} case. The interaction with the phosphates is not stable in either case, but Mg_C's extra charge causes greater electrostatic repulsion, leading to farther distances. The AMBER GAFF2 12-6 simulations show binding of Mg_C in only the S113A state, but the Arg61 behavior is not much different in the WT case, likely due to the presence of Mg_C during the early parts of the simulation. In only one run of the AMBER S113A two-Mg^{2+} simulation did Arg61 stay bound to the phosphates the entire time (and one of each S113A and WT simulations with CHARMM). In the GAFF 12-6-4 WT simulations, Arg61 has a greater population of the bound conformation in the two-Mg^{2+} simulations than in the three-Mg^{2+} simulations. This is true of m12-6-4 and OL15 as well (all proteins), although the effect is slightly altered in the OL15 case with the unbound distance less far than for the m12-6-4 case. In the product state, Arg61 binds the PPi phosphates in all the simulations with the m12-6-4 parameters. In the GAFF2 12-6-4 simulations, Arg61 does not sample the unbound conformation in the S113A mutant and is unbound in one run of the WT simulations regardless of starting with two or three Mg^{2+} ions. The behavior is very different with the GAFF2 12-6 ion model, where Arg61 switches readily between bound and unbound conformations in 4 of the 5 simulations for both proteins with three Mg^{2+}. The two-Mg^{2+} cases appear much more stable, with only 1 run showing Arg61 moving away from the PPi phosphates for both proteins.

The Arg61 residue shows strong trend in the DFTB3/MM simulations as well. The presence of Mg_C in the active site reactant state causes Arg61 to rotate away in three of the WT simulations and all five of the S113A runs. Arg61 unbinds in only one of the S113A

simulations with two Mg^{2+}. In the product state, Arg61 stays bound. Mg_C and Arg61 experience electrostatic repulsion in the reactant state, but Arg61 plays a crucial role in stabilizing negative charge when Mg_C is not present or there is greater negative charge in the product state.

Ligand Unbinding Umbrella Sampling

We simulated the unbinding process (m12-6-4 ions) with umbrella sampling. The unbinding pathway (Figure 4**C** in the main text) appears remarkably similar to **B** with base stacking interactions and orientation of the nucleotide. The key difference is that the DNA primer orientation is maintained in umbrella sampling but disrupted in unbiased MD. Further evidence for this being on-pathway for the ligand (un)binding process is the role of S113 in proper DNA duplex primer docking.[44] In the WT m12-6-4 and OL15 WT three-Mg^{2+} simulation, S113 hydrogen bonds to the Thy8 3'OH only when the dATP ligand has shifted. The predominant hydrogen bonding partner of S113 is water and the hydrogen bond network in the active site is perturbed by by loss of the S113 HB donor, which may be why the 3'OH-Mg_A interaction is broken. Disruption occurs when Mg_C is not bound, and according to experiment Mg_C is only bound in the reactant state right before the nucleophilic reaction proceeds. All together, these suggest that the presence of Mg_C stabilizes the reactant state, but this binding is transient. Without Mg_C, active site disruption occurs due to fluctuations in ordered waters; the disruption is greater with the S113A mutation, resulting in shifting of the dATP ligand that appears on pathway to (un)binding. Although the CHARMM simulations also showed active site disruption with the S113A mutation and stabilization by Mg_C, these simulations are qualitatively different in the binding propensity of Mg_C and the roles of key amino acids in the active site. The m12-6-4 simulations are more reasonable based on experimental comparison and the smaller degree of active site disruption.

The unbinding of the dATP ligand (Figure S.5, top) shows differences in binding/unbinding free energy when only the ligand unbinds or when Mg_B travels with it. These differences

show that more binding free energy is released when the dATP ligand binds with Mg_B (-177.2 $kcal \cdot mol^{-1}$ compared to -127.7 $kcal \cdot mol^{-1}$), in part because of the favorable chelating interactions between residues of the DED motif and the Mg_B ion. These binding PMFs are barrier-less, but do not include a Jacobian term which could add an entropic barrier to the overall free energy profile. That being said, this correction would affect both PMF curves and does not affect the differences between them. The two curves here are two edges of a thermodynamic cycle which can be completed by considering the following other two edges: the binding of Mg_B to the protein without ligand and binding of Mg_B to dATP in the bulk. The approximately 49.8 $kcal \cdot mol^{-1}$ difference between the two PMF curves must therefore be the sum of these two un-estimated parts of the thermodynamic cycle, suggesting very strong binding interactions between the protein and Mg_B. This suggests that dATP ligand binding is likely to occur with Mg^{2+}, but there is no kinetic benefit for this, just a thermodynamic one.

For product pyrophosphate unbinding, we started from the product well of the Mg_C migration PMF, and test the cases where either just Mg_C or also Mg_B dissociate with the PP_i. As is the case with dATP binding/unbinding with Mg_B, unbinding of PP_i with both Mg_C and Mg_B is more unfavorable (91.5 $kcal \cdot mol^{-1}$) compared to just unbinding with Mg_C (44.5 $kcal \cdot mol^{-1}$). Additionally, the PMFs are also barrier-less as was seen for the dATP ligand binding. The difference between these values is again the difference between the (product) protein/DNA complex binding Mg_B and the PP_i+Mg_C complex binding with Mg_B in the bulk. This difference is similar at 47.0 $kcal \cdot mol^{-1}$ to the difference seen with the dATP ligand binding, perhaps suggesting a similar magnitude trade off of Mg_B binding to the protein and bulk-solvated ligand in both cases. The unbinding of PP_i is less unfavorable compared to the dATP ligand.

We see no kinetic preference for one or two Mg^{2+} ions leaving with PP_i since the reactions are essentially barrier less from our potential of mean force. The difference would therefore only be thermodynamic. This is in contrast to other works (on DNAP[5] with metadynamics

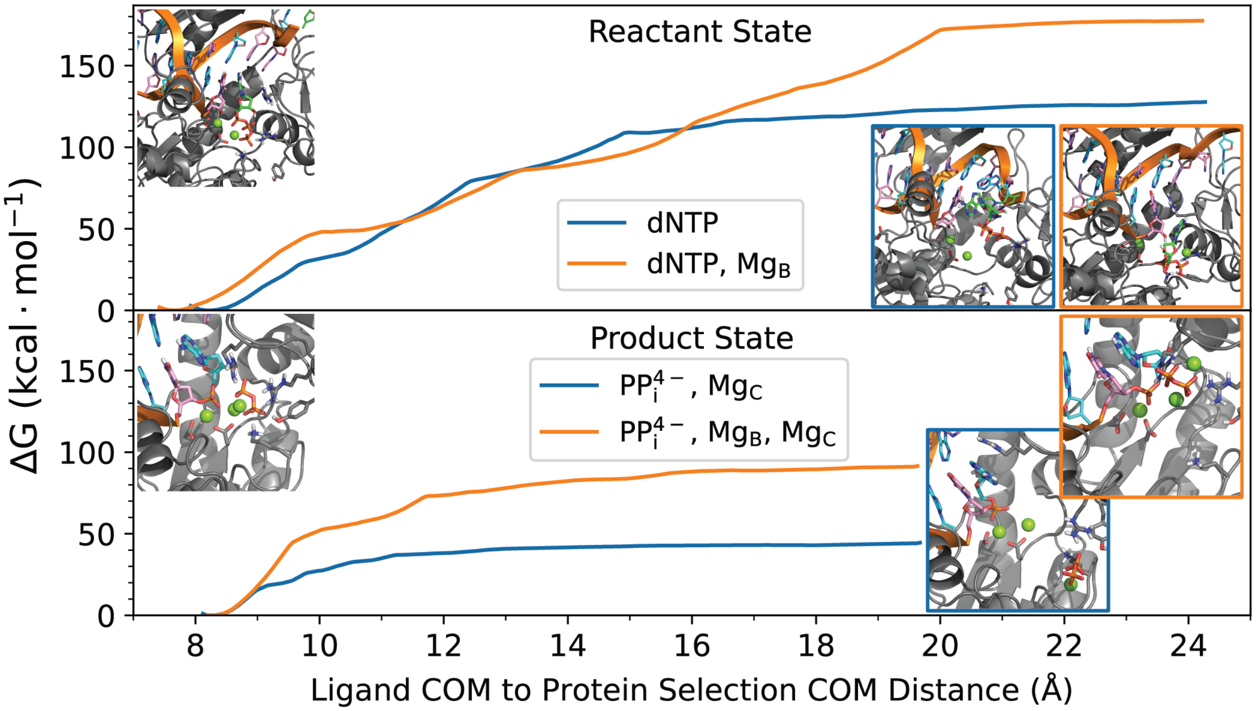

Figure S.5: Potential of mean force for dATP and PP_i unbinding—Top: in the reactant state (dATP) with either zero or one Mg^{2+} ions (started from the two Mg^{2+} case). Bottom: in the product state (PP_i) with one or two Mg^{2+} ions (started from the three Mg^{2+} case). Snapshots on the left show the bound ligand (shared structure per state for both curves) and on the right, structures from the last umbrella window (colored with borders according to the legends).

or in RNA polymerases[48–50] with Markov state modeling) that show there is a kinetic barrier to PP_i dissociation and that the unbound state has lower free energy than the bound state. Since all of these methods use physical unbinding events, there is a challenge to know the correct binding free energy value that would be obtained from alchemical methods. These differences could perhaps be due to the limitations of performing umbrella sampling along only one unbinding pathway.[51] Additionally, we have used a simple collective variable to sample the unbinding process (distance between the center of mass of selected protein active site residues and the center of mass of the ligand). This collective variable is likely insufficient to capture important relaxation associated both with protein conformational changes, and in the case of dATP ligand, reorganization of the floppy ligand itself. An additional challenge is that the ligand and active site are both highly charged. All these considerations might

explain the very large magnitudes of the free energy changes observed from the umbrella sampling simulations.

However, we also note the shape of our PMFs without a true barrier for both product and reactant binding appear similar to the shape of curves from various other works on diverse binding partners.[51–53] Of particular note is the work of Atis *et al.* (2017) which showed PP_i release from HIV reverse transcriptases is barrier-less with milestoning although the free energy of unbinding with one Mg^{2+} ion is much less (although still positive) than we have calculated here. Thus, although our results differ from previous works and the limitations of umbrella sampling are known, we believe our PMF calculations for ligand binding are sufficient to answer structural questions about residue interactions along the unbinding pathway and compare the effect of the number of Mg^{2+} ions that leave with the ligand.

QM Benchmark of Sugar Puckering Highlights DFTB3's Shortcomings

DFTB3 is not reliable for sugar puckering.[23,24] To benchmark this, we generated three models for sugar puckering as shown in Figure S.7. These models are either small, intermediate, or large. The smallest is a simple tetrahydrofuran ring with either one or two hydroxyl groups, representing the groups found on deoxyribose and ribose sugars respectively. The intermediate model adds the nitrogenous base (here thymidine), and the largest model adds the C5 sugar carbon and has a methyl phosphate group attached (representing a full nucleotide as seen in Figure S.6). Because the 3'OH of the primer strand must become deprotonated for nucleophilic attack, we also review the effect of deprotonation on sugar puckering. These full models can be seen in Figure S.6. The structures of these models at the DFTB3 level were subject to single point energy calculations with several methods: MP2, B3LYP-D3(BJ), SCAN0+VV10, and REVSCAN0+VV10.

As can be seen in the simplest models (Figure S.10 and Figure S.11), DFTB3's sugar

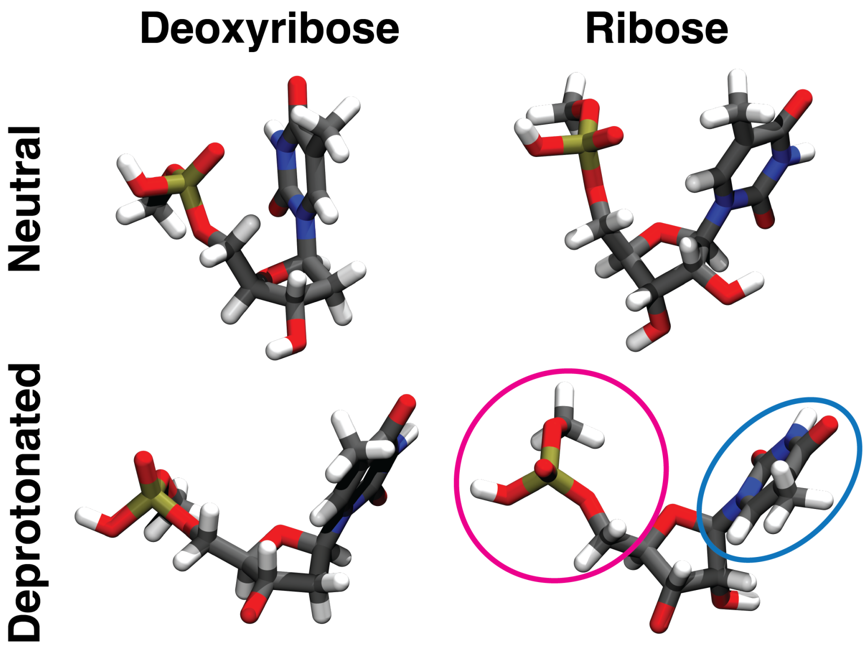

Figure S.6: Structures of the lowest MP2 energy sugar puckering conformation from the largest model system, (deoxy)thymidine methyl-mono-phosphate. Note the differences in sugar puckering between the neutral and deprotonated forms of both the deoxyribose and ribose sugars. The deprotonated confirmation are very similar between the two sugars in the deprotonated state, but different in the neutral state. The ovals on the lower right structure are used to highlight the regions removed for the intermediate model (pink) and then for the small model (blue) (Figure S.7). Atoms are colored according to: C=grey, H=white, N=blue, O=red, P=gold.

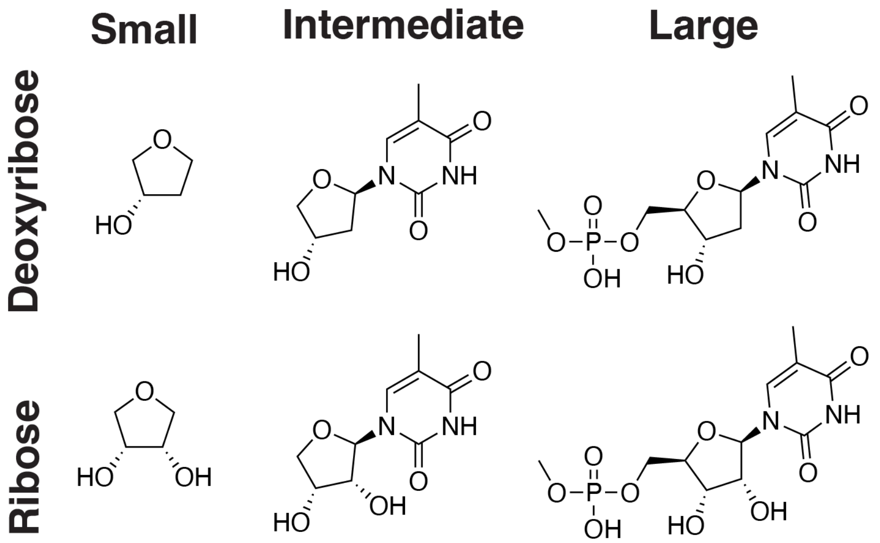

Figure S.7: Scheme depicting the sugar pucker model systems (ribose and deoxyribose) of varying sizes (small, intermediate, and large) used in this work to assess the sugar puckering capability of DFTB3 as compared to *ab initio* methods like MP2 and DFT.

puckering is too flat and minima are not correctly reproduced. For instance, for the deoxyribose case in Figure S.10, the neutral case shows a single well in all *ab initio* methods at the due west direction (270°). DFTB3 on the other hand predicts a very broad well that takes up the whole upper right quadrant of the graph. This well is the same in the deprotonated case with DFTB3, although perhaps slightly more localized at 0°(on the graphs, as would be typical for polar coordinates, east is 0°, north is 90°, west is 180°, and south is 270°). In the *ab initio* methods, the western well is still the minimum but there is now a second well at 0° that just happens to coincide with DFTB3. The REVSCAN0+VV10 graph compares best with MP2 in the estimated puckering barrier between the wells on both potential directions around the puckering polar coordinate. The second 0° wells of the DFT methods are slightly lower than the energy of MP2 but below 1 $\text{kcal} \cdot \text{mol}^{-1}$different. For the ribose case in Figure S.11, both neutral and deprotonated models have two wells in the *ab initio* methods. DFTB3 has what would be better described as a metastable energy minimum for the well at 270°. However, there is no barrier for this transition in DFTB3 in either case. For the neutral model, MP2 predicts a barrier slightly above 4 $\text{kcal} \cdot \text{mol}^{-1}$around the upper

half (slightly above 5 $\text{kcal}\cdot\text{mol}^{-1}$for the bottom half). In the deprotonated case, the upper barrier is ≈ 4.5 $\text{kcal}\cdot\text{mol}^{-1}$ (and about 7 for the bottom half). In this case, SCAN0+VV10 best matches MP2.

The intermediate sugar puckering models display a similar trend. There is one minimum in the neutral deoxyribose scans at around 10° which roughly corresponds to the C3'-endo configuration (Figure S.12, top). This is true for all the methods, but DFTB3 does not have a barrier in the center of the graph, nor does it match the correct barrier for pucker conformations when completing a full revolution. The deprotonated cases have a small metastable state near 180° in addition to a similar well as the neutral case at 0°. This metastable state is captured by the DFT methods, but none match the barrier or state energy exactly, with B3LYP+D3(BJ) being the closest. DFTB3 once again lacks any barrier in the center of the graph, which represents a flat ring. In the ribose case (Figure S.13), DFTB3 does a better (but not great) job. It correctly identifies two minima for the neutral and deprotonated cases. The second well near 180° in the neutral case is not stabilized enough and the barrier is too low to inter-convert between them. Additionally, the center lacks a significant barrier as needed. In the deprotonated case, DFTB3 actually matches well with the B3LYP-D3(BJ) method, but both underestimate the stabilization of the higher energy well of MP2. In the neutral case, the MP2 results are best matched by the REVSCAN0+VV10 dispersion, and in the deprotonated case by B3LYP-D3(BJ).

In the largest sugar puckering models, DFTB3 occasionally picks up on key regions of interest but with false positives and negatives. For instance, in the deoxyribose puckering model of Figure S.8, the neutral MP2 calculations show a major minimum near 270°, and a minor one at 45°. DFTB3 and B3LYP+D3(BJ) both show three minima, two of which match the locations of MP2. The third minimum is in the south-west corner of the graph. All of these minima are similar in energetics to each other and but the puckering rotation barrier is too low (worse in DFTB3)—these features are remarkably incorrect. In the deprotonated case, DFTB3 says there should be one minimum. This is the global minimum of MP2 but

MP2 now has three energy wells in total. The transition energy between wells along the north region of the graph is best matched with B3LYP+D3(BJ) but this DFT method misses the southern most well. The best match here is likely SCAN0+VV10 dispersion although it has a slightly too high transition barrier—SCAN0+VV10 is also nearly matched in quality by REVSCAN0+VV10.

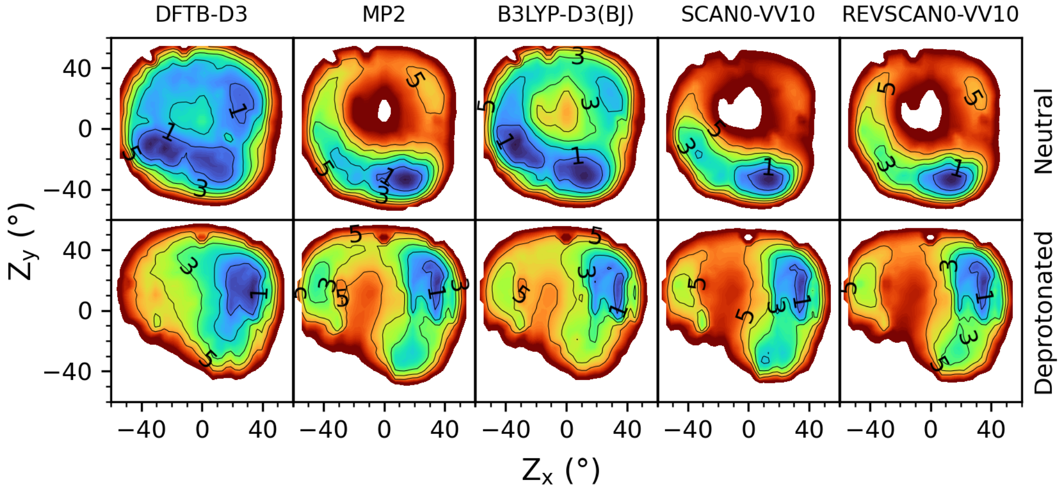

Figure S.8: Potential energy scans from the largest deoxyribose model system, deoxythymidine methyl-mono-phosphate (in $\text{kcal} \cdot \text{mol}^{-1}$) of sugar puckering at the DFTB3 level and single point energies of those structures from higher level *ab initio* calculations including MP2 and several DFT functionals. Contours are colored from 0 (blue) to 7 (red) with contour lines every 1 $\text{kcal} \cdot \text{mol}^{-1}$ and every other contour line labeled with the energy. Sugar pucker pseudo-rotation angles can be extracted as follows—east: 0°, north: 90°, west: 180°, and south: 270°.

In the ribose model (Figure S.9), DFTB3 performs decently for the deprotonated case. It has two wells just like MP2 and they share the global minima. The barrier to transition to the other well is about right but the second well is not stable enough. B3LYP+D3(BJ) gets the transition barrier correct compared to MP2. All the DFT methods and second well energy location right but do not predict it to be stable enough compared to MP2. In the neutral case, DFTB3 has two minima that are similar in energy. The south-west minimum is close but slightly off in location, but the other (north-east) is correct in location. DFTB3 lacks an energy minimum in the south-east section, in part because although the energy is

right in that region, the transitions between wells are so shallow that this location on the graph does not register as a true minimum. MP2 is best matched by the REVSCAN0+VV10 functional, although SCAN0+VV10 is not far behind in quality. B3LYP+D3(BJ) does not capture the energy of the local-minimum appropriately and is too low in transition barrier height.

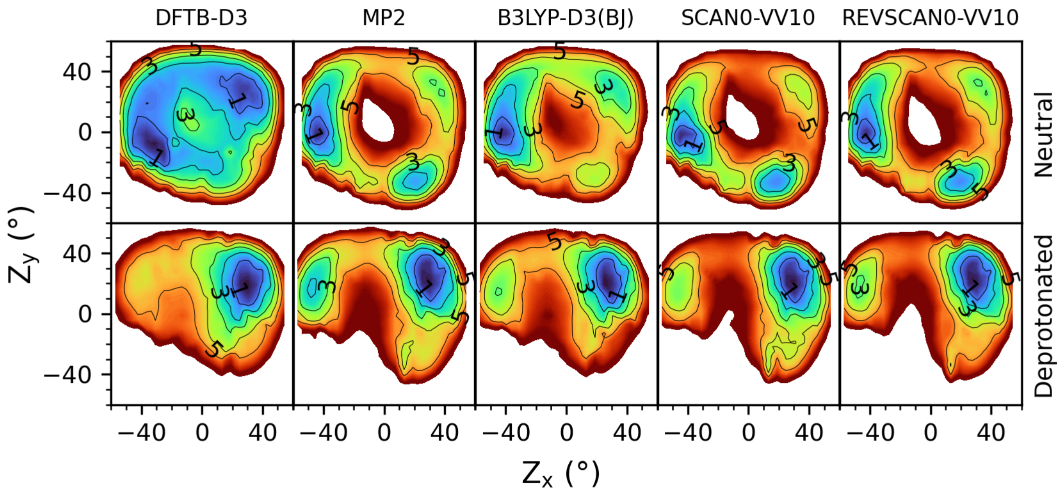

Figure S.9: Potential energy scans from the largest ribose model system, thymidine methyl-mono-phosphate (in kcal · mol^{-1}) of sugar puckering at the DFTB3 level and single point energies of those structures from higher level *ab initio* calculations including MP2 and several DFT functionals. Contours are colored from 0 (blue) to 7 (red) with contour lines every 1 kcal · mol^{-1} and every other contour line labeled with the energy. Sugar pucker pseudo-rotation angles can be extracted as follows—east: 0°, north: 90°, west: 180°, and south: 270°.

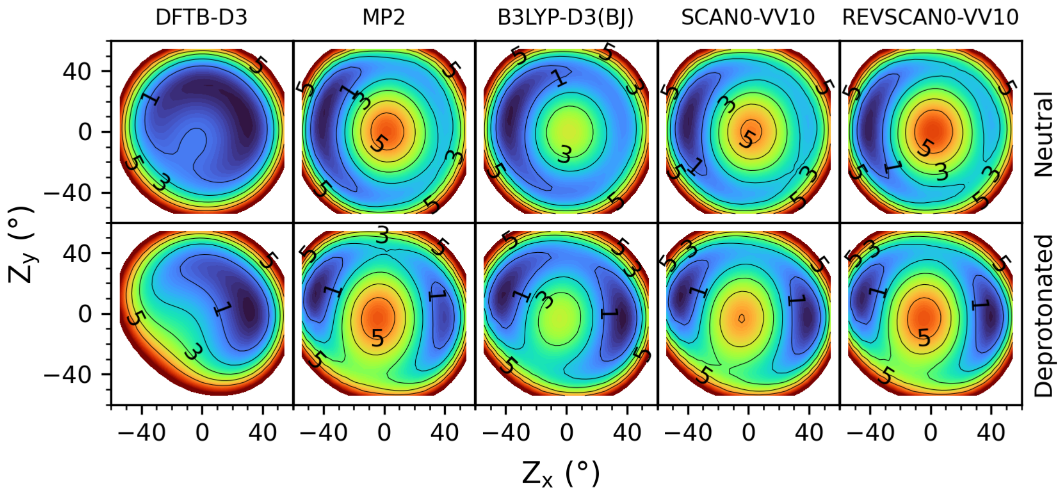

Figure S.10: Potential energy scans from the smallest deoxyribose model system (in kcal · mol^{-1}) of sugar puckering at the DFTB3 level and single point energies of those structures from higher level *ab initio* calculations including MP2 and several DFT functionals. Contours are colored from 0 (blue) to 7 (red) with contour lines every 1 kcal · mol^{-1} and every other contour line labeled with the energy. Sugar pucker pseudo-rotation angles can be extracted as follows—east: 0°, north: 90°, west: 180°, and south: 270°.

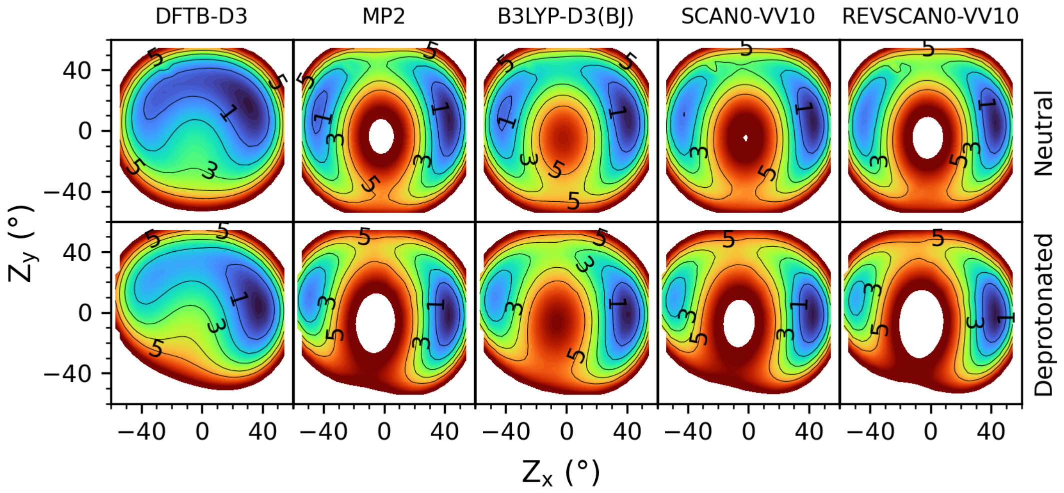

Figure S.11: Potential energy scans from the smallest ribose model system (in kcal · mol^{-1}) of sugar puckering at the DFTB3 level and single point energies of those structures from higher level *ab initio* calculations including MP2 and several DFT functionals. Contours are colored from 0 (blue) to 7 (red) with contour lines every 1 kcal · mol^{-1} and every other contour line labeled with the energy. Sugar pucker pseudo-rotation angles can be extracted as follows—east: 0°, north: 90°, west: 180°, and south: 270°.

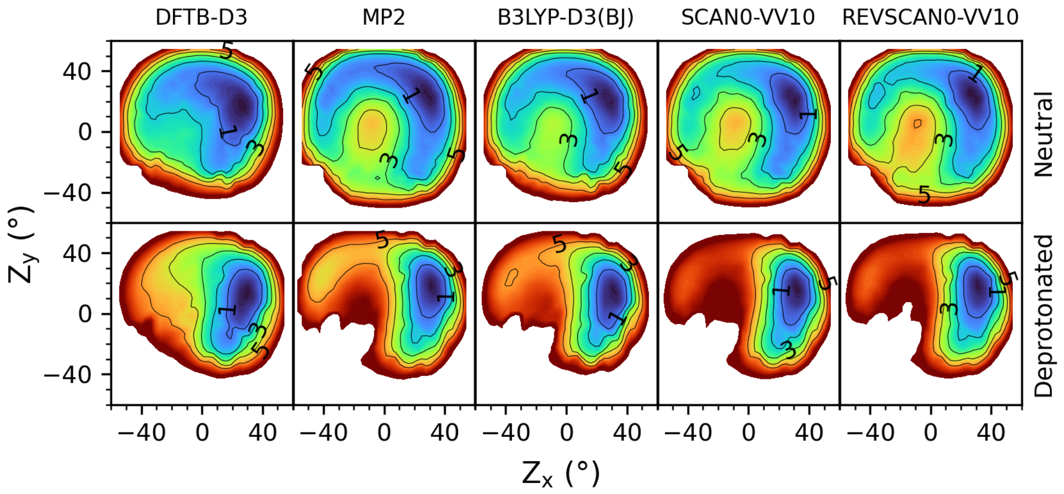

Figure S.12: Potential energy scans from the intermediate deoxyribose model system (in $\text{kcal} \cdot \text{mol}^{-1}$) of sugar puckering at the DFTB3 level and single point energies of those structures from higher level *ab initio* calculations including MP2 and several DFT functionals. Contours are colored from 0 (blue) to 7 (red) with contour lines every 1 $\text{kcal} \cdot \text{mol}^{-1}$ and every other contour line labeled with the energy. Sugar pucker pseudo-rotation angles can be extracted as follows—east: 0°, north: 90°, west: 180°, and south: 270°.

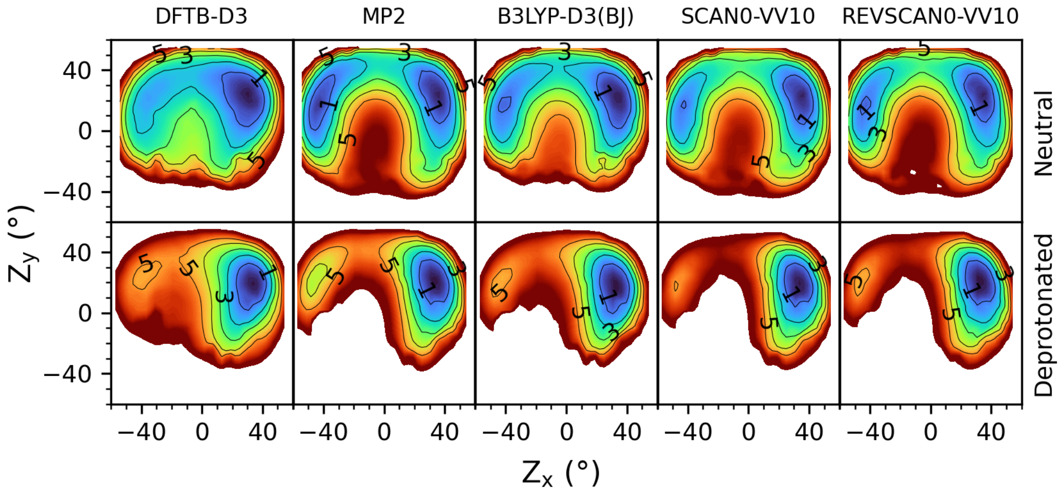

Figure S.13: Potential energy scans from the intermediate ribose model system (in $\text{kcal} \cdot \text{mol}^{-1}$) of sugar puckering at the DFTB3 level and single point energies of those structures from higher level *ab initio* calculations including MP2 and several DFT functionals. Contours are colored from 0 (blue) to 7 (red) with contour lines every 1 $\text{kcal} \cdot \text{mol}^{-1}$ and every other contour line labeled with the energy. Sugar pucker pseudo-rotation angles can be extracted as follows—east: 0°, north: 90°, west: 180°, and south: 270°.

Additional Comments on the CHARMM Mg^{2+} Parameters

As a final note, the CHARMM36 force field default Mg^{2+} parameters have been used extensively,[54] but these are essentially unchanged from the CHARMM27 force field. Careful comparisons have seen challenges in correctly describing the binding of Mg^{2+} to nucleotides (for both CHARMM22[55] and CHARMM27[56] parameters). Allnér *et al.* (2012) concluded that the CHARMM27 unbinding kinetics (barrier height) to Mg^{2+}-water dissociation were too high and parameterized the repulsive Lennard-Jones term to match the experimental value.[56] The dissociation barrier of Mg^{2+} from phosphate was also too high in the original CHARMM27 parameters (21.3 $\text{kcal} \cdot \text{mol}^{-1}$ compared to 12.7 to 13.3 $\text{kcal} \cdot \text{mol}^{-1}$ for experiment) and the above reparameterization showed a better barrier (although still too high at 16.4 $\text{kcal} \cdot \text{mol}^{-1}$) even though it was not directly included as a metric for parameter optimization.[56] This reparameterization also improved the binding potential of mean force by making the close Mg^{2+}-phosphate interactions ≈ 2 $\text{kcal} \cdot \text{mol}^{-1}$ less favored compared to the original parameters at bulk distances.[56] This binding free energy value is still too high when considering the reparameterization of the m12-6-4 ions and the potential of mean force in Panteva *et al.* (2015).[57] It is not surprising that the CHARMM simulations here showed strong binding in our active site, since Mg^{2+} dissociation has an unrealistically high barrier (and a too strong binding free energy). Thus we caution the use of the default CHARMM Mg^{2+} ion parameters since alternatives match key experimental data pertaining to both Mg^{2+}-water and Mg^{2+}-phosphate interactions much better.[56,58]

Equilibration Time Series

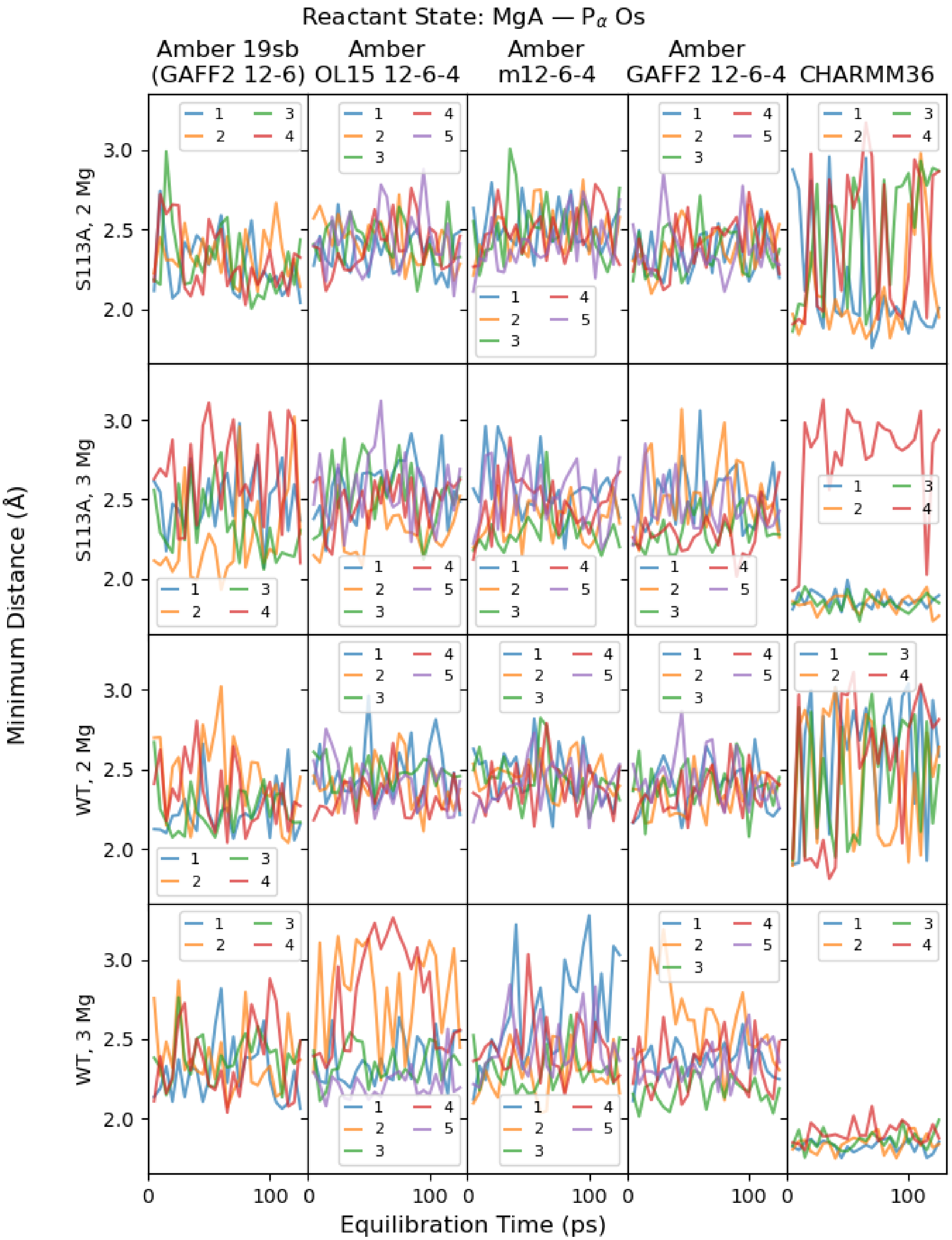

Figure S.14: Equilibration time series for the minimum distance between Mg_A and the phosphate oxygens of P_α in the reactant state for all simulations.

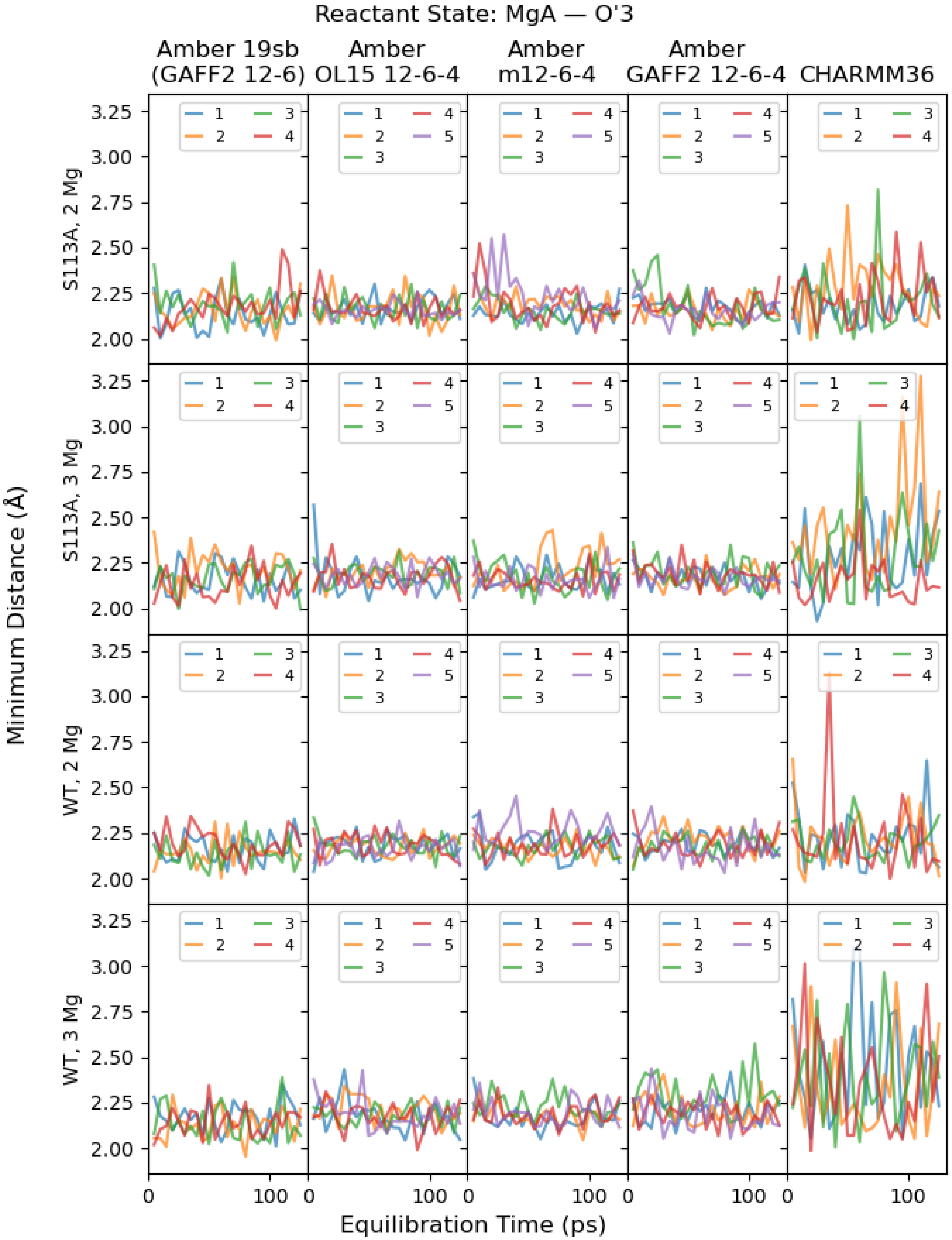

Figure S.15: Equilibration time series for the minimum distance between Mg_A and the 3'O in the reactant state for all simulations.

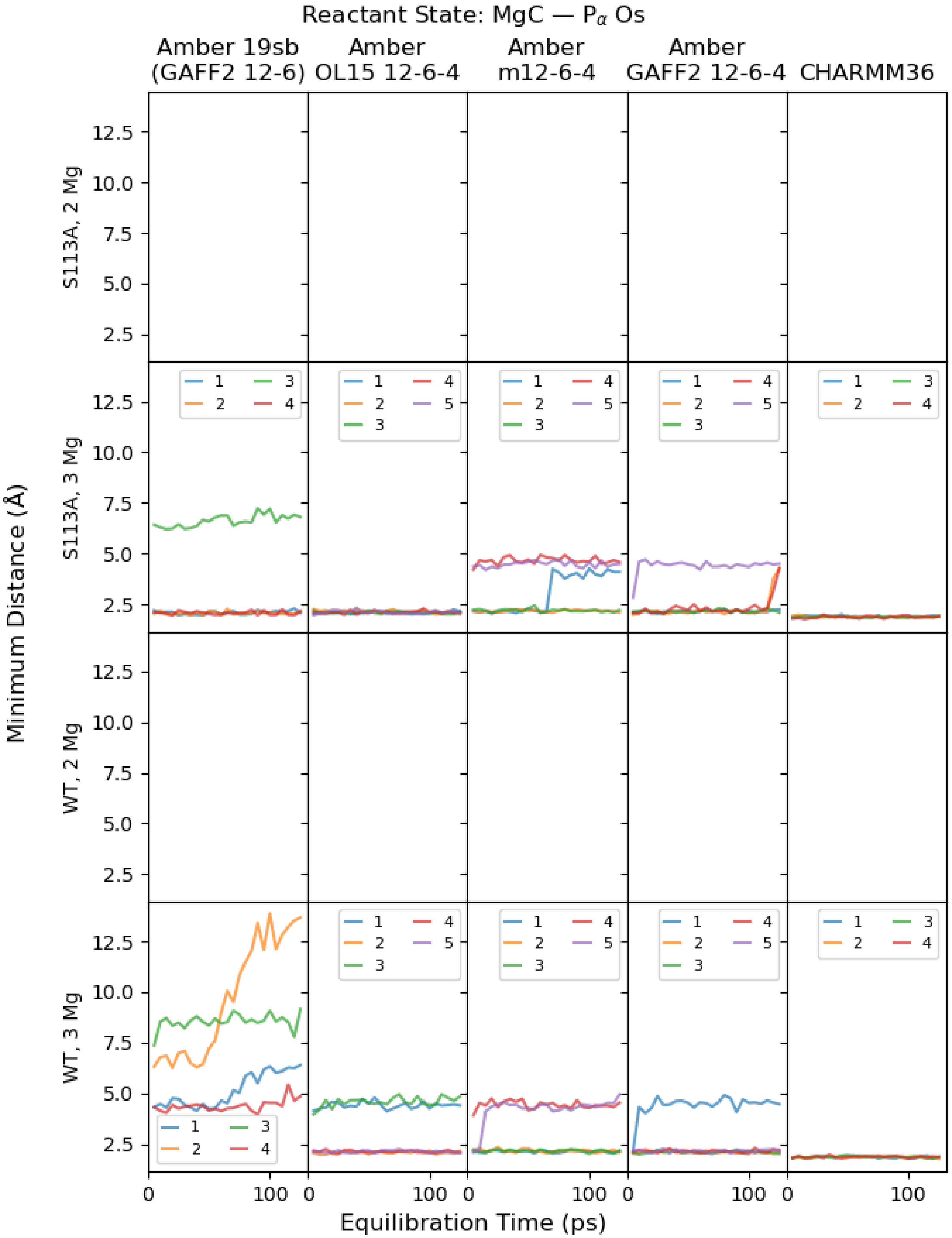

Figure S.16: Equilibration time series for the minimum distance between Mg_C and the phosphate oxygens of P_α in the reactant state for all simulations.

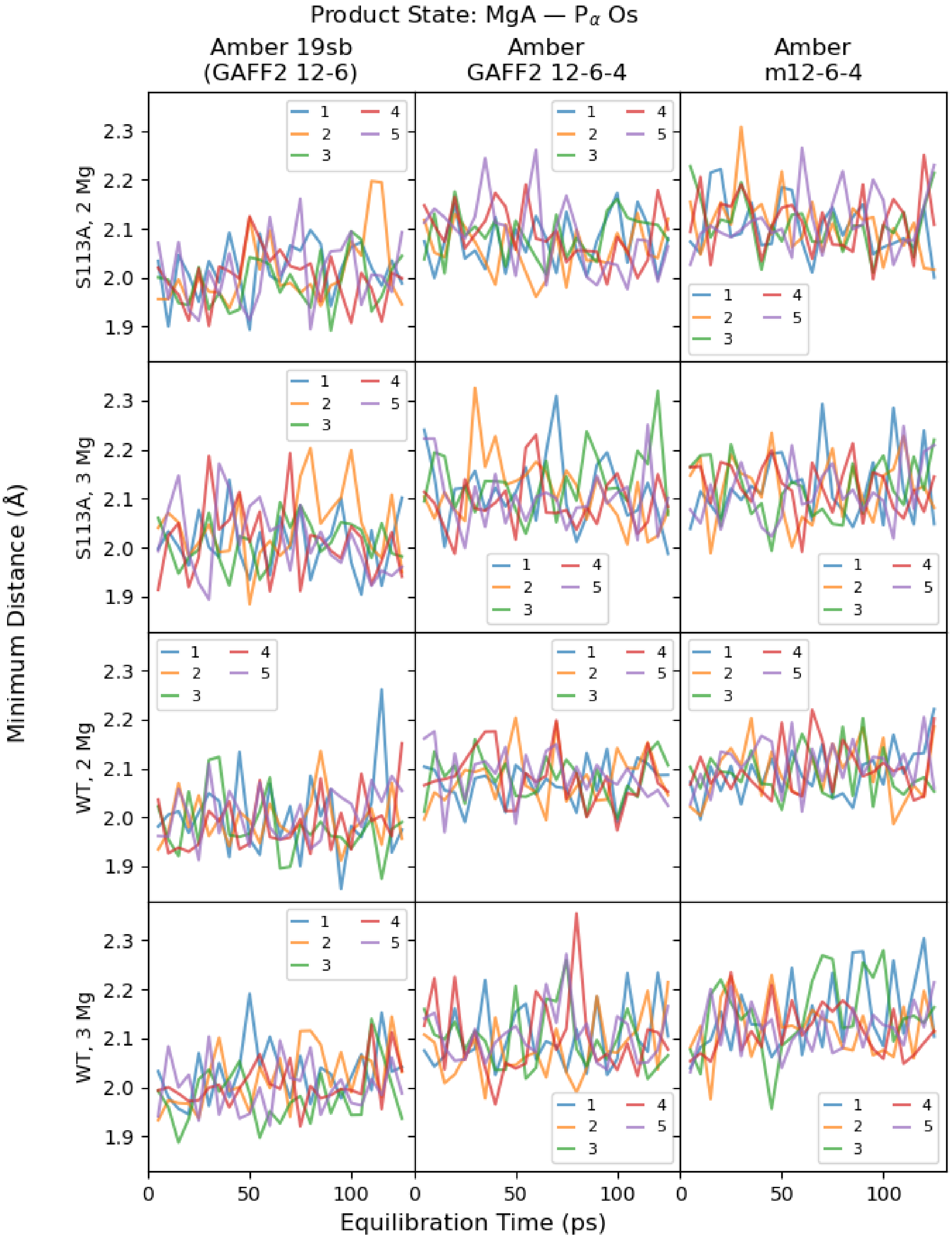

Figure S.17: Equilibration time series for the minimum distance between Mg_A and the phosphate oxygens of P_α in the product state for all simulations.

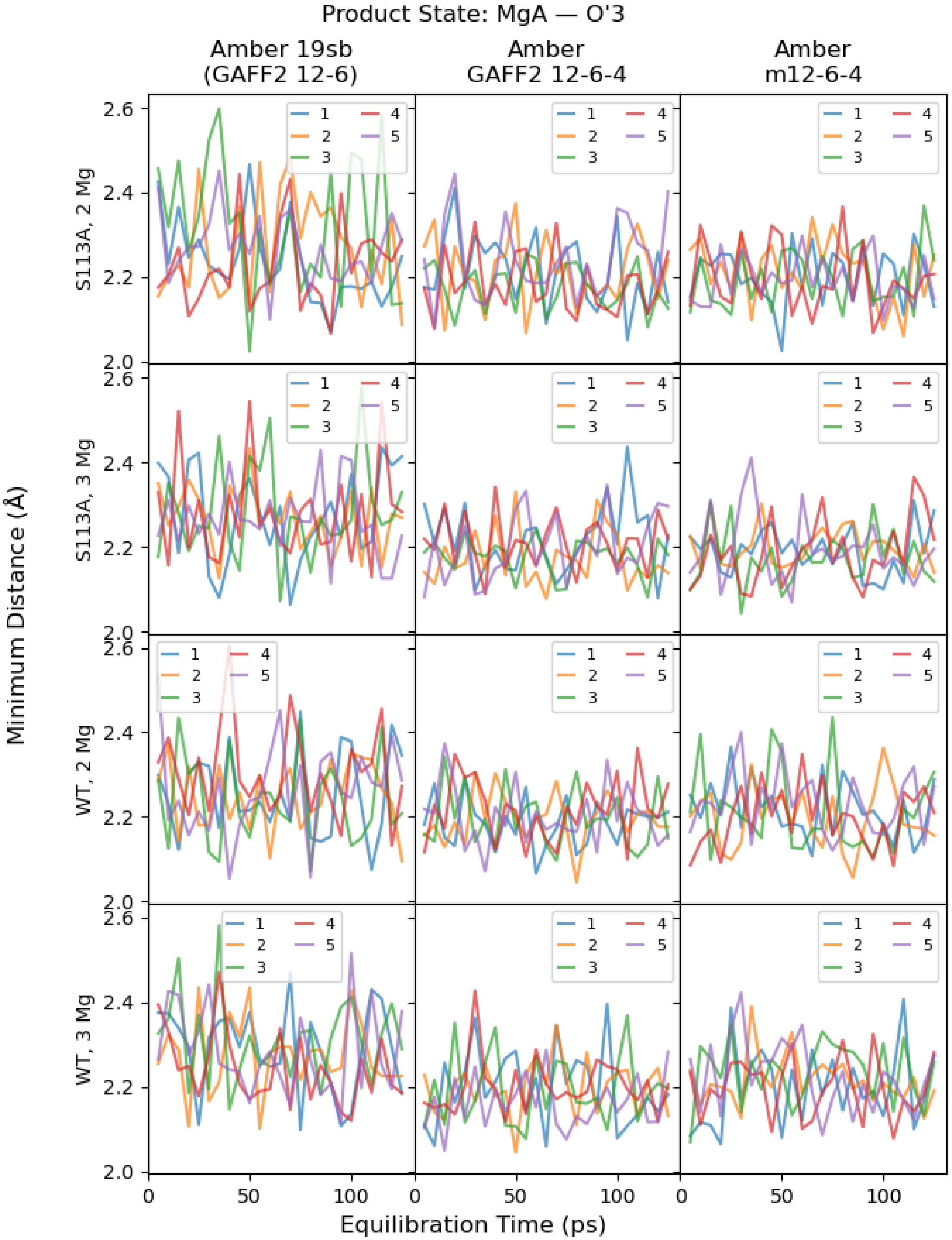

Figure S.18: Equilibration time series for the minimum distance between Mg_A and the 3'O in the product state for all simulations.

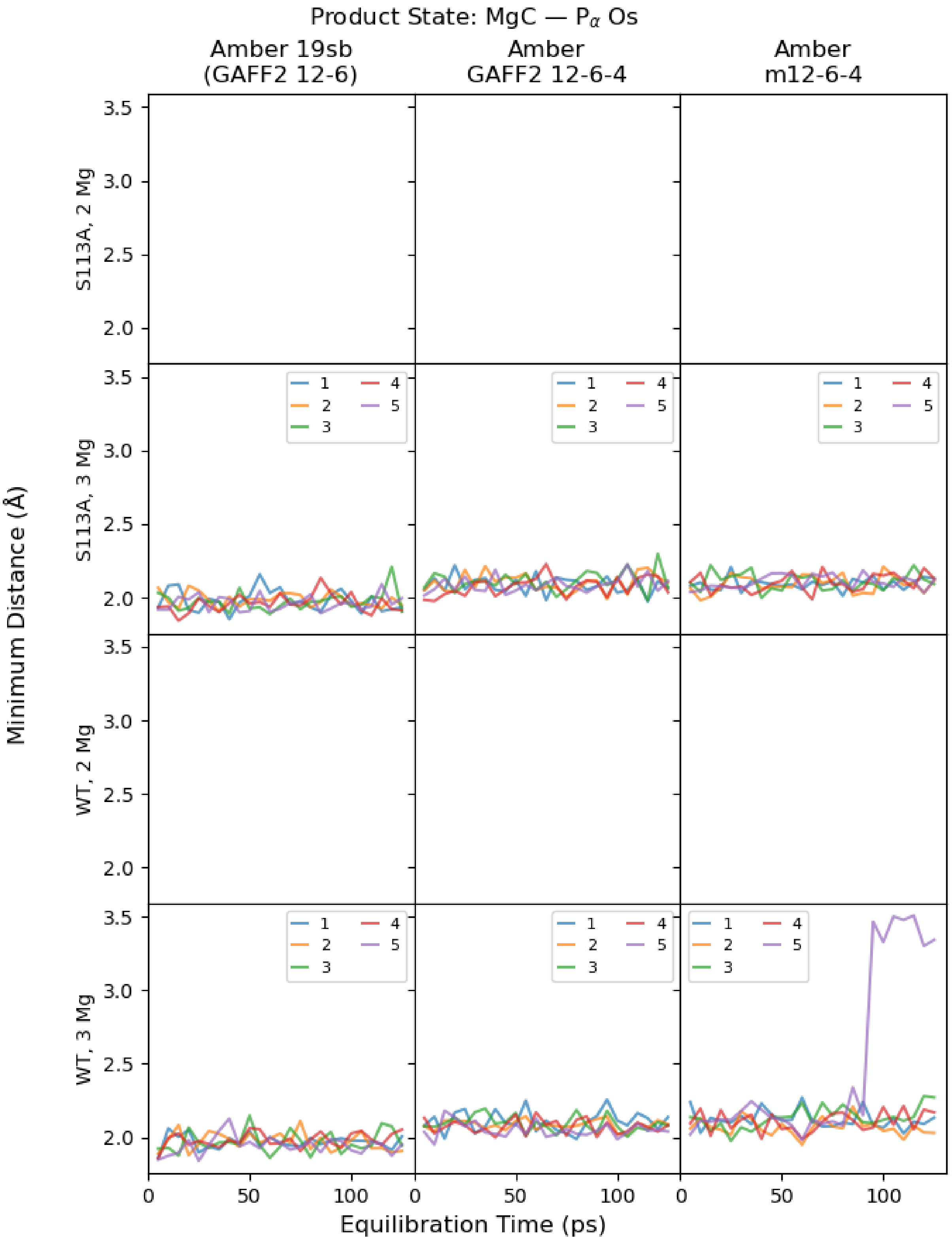

Figure S.19: Equilibration time series for the minimum distance between Mg_C and the phosphate oxygens of P_α in the product state for all simulations.

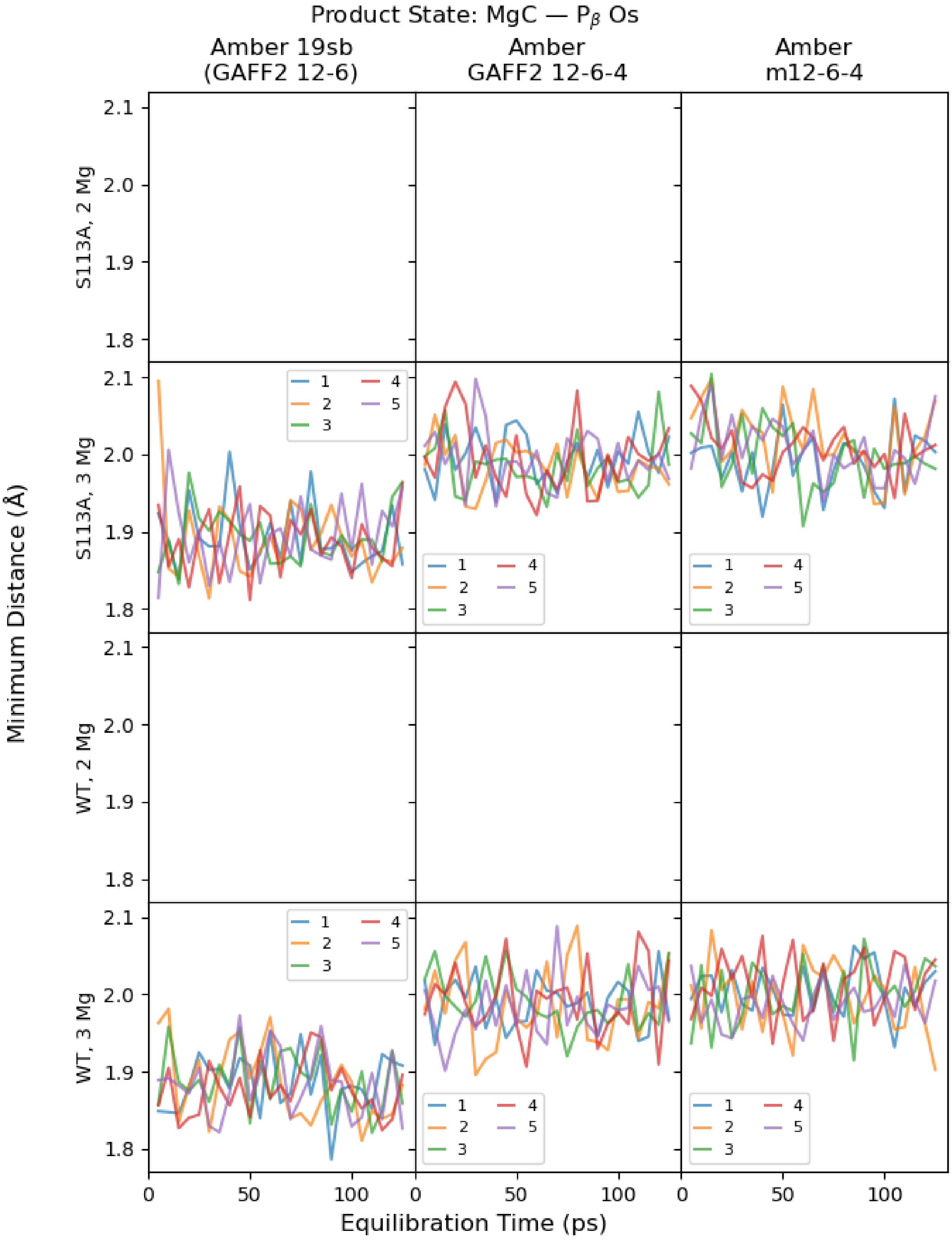

Figure S.20: Equilibration time series for the minimum distance between Mg_C and the phosphate oxygens of P_β in the product state for all simulations.

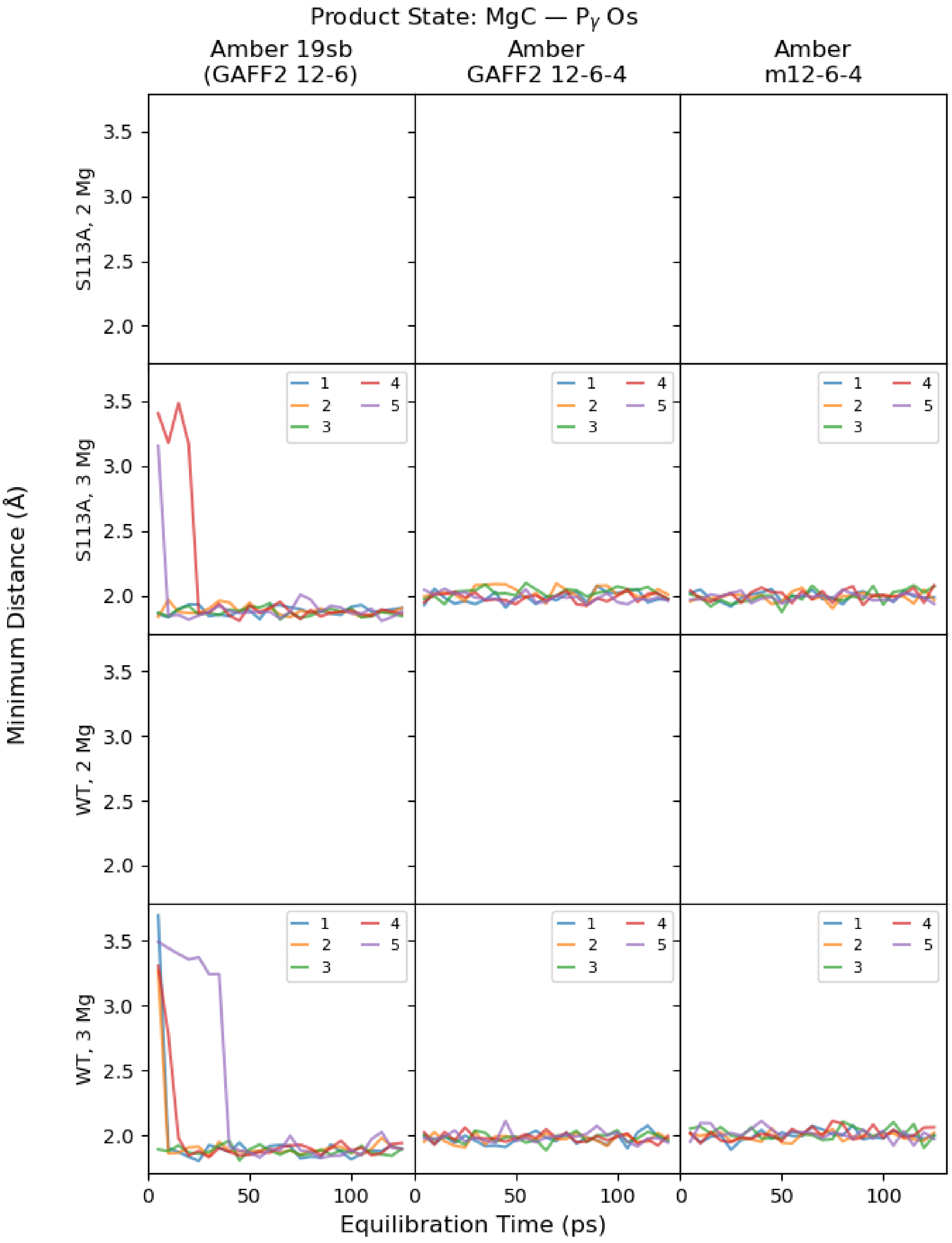

Figure S.21: Equilibration time series for the minimum distance between Mg_C and the phosphate oxygens of P_γ in the product state for all simulations.

References

(1) D.A. Case, H.M. Aktulga, K. Belfon, I.Y. Ben-Shalom, J.T. Berryman, S.R. Brozell, D.S. Cerutti, T.E. Cheatham, III, G.A. Cisneros, V.W.D. Cruzeiro, T.A. Darden, R.E. Duke, G. Giambasu, M.K. Gilson, H. Gohlke, A.W. Goetz, R. Harris, S. Izadi, S.A. Izmailov, K. Kasavajhala, M.C. Kaymak, E. King, A. Kovalenko, T. Kurtzman, T.S. Lee, S. LeGrand, P. Li, C. Lin, J. Liu, T. Luchko, R. Luo, M. Machado, V. Man, M. Manathunga, K.M. Merz, Y. Miao, O. Mikhailovskii, G. Monard, H. Nguyen, K.A. O'Hearn, A. Onufriev, F. Pan, S. Pantano, R. Qi, A. Rahnamoun, D.R. Roe, A. Roitberg, C. Sagui, S. Schott-Verdugo, A. Shajan, J. Shen, C.L. Simmerling, N.R. Skrynnikov, J. Smith, J. Swails, R.C. Walker, J. Wang, J. Wang, H. Wei, R.M. Wolf, X. Wu, Y. Xiong, Y. Xue, D.M. York, S. Zhao, and P.A. Kollman (2022), Amber 2022, University of California, San Francisco.

(2) Shirts, M. R.; Chodera, J. D. Statistically Optimal Analysis of Samples from Multiple Equilibrium States. *J. Chem. Phys.* **2008**, *129*, 124105.

(3) Chodera, J. D. A Simple Method for Automated Equilibration Detection in Molecular Simulations. *J. Chem. Theory Comput.* **2016**, *12*, 1799–1805.

(4) Shirts, M. R.; Ferguson, A. L. Statistically Optimal Continuous Free Energy Surfaces from Biased Simulations and Multistate Reweighting. *J. Chem. Theory Comput.* **2020**, *16*, 4107–4125.

(5) Genna, V.; Gaspari, R.; Dal Peraro, M.; De Vivo, M. Cooperative Motion of a Key Positively Charged Residue and Metal Ions for DNA Replication Catalyzed by Human DNA Polymerase-η. *Nucleic Acids Res.* **2016**, *44*, 2827–2836.

(6) Roston, D.; Demapan, D.; Cui, Q. Extensive Free-Energy Simulations Identify Water as the Base in Nucleotide Addition by DNA Polymerase. *Proc. Natl. Acad. Sci. U. S. A.* **2019**, *116*, 25048–25056.

(7) Elstner, M.; Porezag, D.; Jungnickel, G.; Elsner, J.; Haugk, M.; Frauenheim, Th.; Suhai, S.; Seifert, G. Self-Consistent-Charge Density-Functional Tight-Binding Method for Simulations of Complex Materials Properties. *Phys. Rev. B* **1998**, *58*, 7260–7268.

(8) Gaus, M.; Cui, Q.; Elstner, M. DFTB3: Extension of the Self-Consistent-Charge Density-Functional Tight-Binding Method (SCC-DFTB). *J. Chem. Theory Comput.* **2011**, *7*, 931–948.

(9) Cui, Q.; Elstner, M.; Kaxiras, E.; Frauenheim, T.; Karplus, M. A QM/MM Implementation of the Self-Consistent Charge Density Functional Tight Binding (SCC-DFTB) Method. *J. Phys. Chem. B* **2001**, *105*, 569–585.

(10) Gaus, M.; Lu, X.; Elstner, M.; Cui, Q. Parameterization of DFTB3/3OB for Sulfur and Phosphorus for Chemical and Biological Applications. *J. Chem. Theory Comput.* **2014**, *10*, 1518–1537.

(11) Lu, X.; Gaus, M.; Elstner, M.; Cui, Q. Parametrization of DFTB3/3OB for Magnesium and Zinc for Chemical and Biological Applications. *J. Phys. Chem. B* **2015**, *119*, 1062–1082.

(12) Grimme, S.; Antony, J.; Ehrlich, S.; Krieg, H. A Consistent and Accurate Ab Initio Parametrization of Density Functional Dispersion Correction (DFT-D) for the 94 Elements H-Pu. *J. Chem. Phys.* **2010**, *132*, 154104.

(13) Grimme, S.; Ehrlich, S.; Goerigk, L. Effect of the Damping Function in Dispersion Corrected Density Functional Theory. *J. Comput. Chem.* **2011**, *32*, 1456–1465.

(14) MacKerell, A. D. et al. All-Atom Empirical Potential for Molecular Modeling and Dynamics Studies of Proteins. *J. Phys. Chem. B* **1998**, *102*, 3586–3616.

(15) MacKerell, A. D.; Feig, M.; Brooks, C. L. Improved Treatment of the Protein Backbone in Empirical Force Fields. *J. Am. Chem. Soc.* **2004**, *126*, 698–699.

(16) Jorgensen, W. L.; Chandrasekhar, J.; Madura, J. D.; Impey, R. W.; Klein, M. L. Comparison of Simple Potential Functions for Simulating Liquid Water. *J. Chem. Phys.* **1983**, *79*, 926–935.

(17) Rowley, C. N.; Roux, B. The Solvation Structure of Na^+ and K^+ in Liquid Water Determined from High Level Ab Initio Molecular Dynamics Simulations. *J. Chem. Theory Comput.* **2012**, *8*, 3526–3535.

(18) Brooks, B. R.; Bruccoleri, R. E.; Olafson, B. D.; States, D. J.; Swaminathan, S.; Karplus, M. CHARMM: A Program for Macromolecular Energy, Minimization, and Dynamics Calculations. *J. Comput. Chem.* **1983**, *4*, 187–217.

(19) Brooks, B. R. et al. CHARMM: The Biomolecular Simulation Program. *J. Comput. Chem.* **2009**, *30*, 1545–1614.

(20) Hwang, W. et al. CHARMM at 45: Enhancements in Accessibility, Functionality, and Speed. *J. Phys. Chem. B* **2024**, *128*, 9976–10042.

(21) Ryckaert, J.-P.; Ciccotti, G.; Berendsen, H. J. Numerical Integration of the Cartesian Equations of Motion of a System with Constraints: Molecular Dynamics of n-Alkanes. *J. Comput. Phys.* **1977**, *23*, 327–341.

(22) Kim, S.; Lee, J.; Jo, S.; Brooks III, C. L.; Lee, H. S.; Im, W. CHARMM-GUI Ligand Reader and Modeler for CHARMM Force Field Generation of Small Molecules. *J. Comput. Chem.* **2017**, *38*, 1879–1886.

(23) Huang, M.; Giese, T. J.; Lee, T.-S.; York, D. M. Improvement of DNA and RNA Sugar Pucker Profiles from Semiempirical Quantum Methods. *J. Chem. Theory Comput.* **2014**, *10*, 1538–1545.

(24) Huang, M.; Dissanayake, T.; Kuechler, E.; Radak, B. K.; Lee, T.-S.; Giese, T. J.;

York, D. M. A Multidimensional B-Spline Correction for Accurate Modeling Sugar Puckering in QM/MM Simulations. *J. Chem. Theory Comput.* **2017**, *13*, 3975–3984.

(25) Kussmann, J.; Ochsenfeld, C. Pre-Selective Screening for Matrix Elements in Linear-Scaling Exact Exchange Calculations. *J. Chem. Phys.* **2013**, *138*, 134114.

(26) Kussmann, J.; Ochsenfeld, C. Preselective Screening for Linear-Scaling Exact Exchange-Gradient Calculations for Graphics Processing Units and General Strong-Scaling Massively Parallel Calculations. *J. Chem. Theory Comput.* **2015**, *11*, 918–922.

(27) Kussmann, J.; Ochsenfeld, C. Hybrid CPU/GPU Integral Engine for Strong-Scaling Ab Initio Methods. *J. Chem. Theory Comput.* **2017**, *13*, 3153–3159.

(28) Laqua, H.; Kussmann, J.; Ochsenfeld, C. Accelerating Seminumerical Fock-exchange Calculations Using Mixed Single- and Double-Precision Arithmethic. *J. Chem. Phys.* **2021**, *154*, 214116.

(29) Laqua, H.; Thompson, T. H.; Kussmann, J.; Ochsenfeld, C. Highly Efficient, Linear-Scaling Seminumerical Exact-Exchange Method for Graphic Processing Units. *J. Chem. Theory Comput.* **2020**, *16*, 1456–1468.

(30) Thompson, T. H.; Ochsenfeld, C. Integral Partition Bounds for Fast and Effective Screening of General One-, Two-, and Many-Electron Integrals. *J. Chem. Phys.* **2019**, *150*, 044101.

(31) Treutler, O.; Ahlrichs, R. Efficient Molecular Numerical Integration Schemes. *J. Chem. Phys.* **1995**, *102*, 346–354.

(32) Laqua, H.; Kussmann, J.; Ochsenfeld, C. An Improved Molecular Partitioning Scheme for Numerical Quadratures in Density Functional Theory. *J. Chem. Phys.* **2018**, *149*, 204111.

(33) Becke, A. D. Density-Functional Exchange-Energy Approximation with Correct Asymptotic Behavior. *Phys. Rev. A* **1988**, *38*, 3098–3100.

(34) Becke, A. D. Density-functional Thermochemistry. III. The Role of Exact Exchange. *J. Chem. Phys.* **1993**, *98*, 5648–5652.

(35) Lee, C.; Yang, W.; Parr, R. G. Development of the Colle-Salvetti Correlation-Energy Formula into a Functional of the Electron Density. *Phys. Rev. B* **1988**, *37*, 785–789.

(36) Grimme, S.; Hansen, A.; Brandenburg, J. G.; Bannwarth, C. Dispersion-Corrected Mean-Field Electronic Structure Methods. *Chem. Rev. (Washington, DC, U. S.)* **2016**, *116*, 5105–5154.

(37) Hui, K.; Chai, J.-D. SCAN-based Hybrid and Double-Hybrid Density Functionals from Models without Fitted Parameters. *J. Chem. Phys.* **2016**, *144*, 044114.

(38) Vydrov, O. A.; Van Voorhis, T. Nonlocal van Der Waals Density Functional: The Simpler the Better. *J. Chem. Phys.* **2010**, *133*, 244103.

(39) Brandenburg, J. G.; Bates, J. E.; Sun, J.; Perdew, J. P. Benchmark Tests of a Strongly Constrained Semilocal Functional with a Long-Range Dispersion Correction. *Phys. Rev. B* **2016**, *94*, 115144.

(40) Mezei, P. D.; Csonka, G. I.; Kállay, M. Simple Modifications of the SCAN Meta-Generalized Gradient Approximation Functional. *J. Chem. Theory Comput.* **2018**, *14*, 2469–2479.

(41) Møller, Chr.; Plesset, M. S. Note on an Approximation Treatment for Many-Electron Systems. *Phys. Rev.* **1934**, *46*, 618–622.

(42) Foloppe, N.; MacKerell, A. D. Conformational Properties of the Deoxyribose and Ribose Moieties of Nucleic Acids: A Quantum Mechanical Study. *J. Phys. Chem. B* **1998**, *102*, 6669–6678.

(43) Frisch, M. J. et al. Gaussian 16 Revision C.01. 2016; Gaussian Inc. Wallingford CT.

(44) Gregory, M. T.; Gao, Y.; Cui, Q.; Yang, W. Multiple Deprotonation Paths of the Nucleophile 3′-OH in the DNA Synthesis Reaction. *Proc. Natl. Acad. Sci. U. S. A.* **2021**, *118*, e2103990118.

(45) Huang, G.-T.; Yu, J.-S. K. Catalytic Roles of Histidine and Arginine in Pyruvate Class II Aldolase: A Perspective from QM/MM Metadynamics. *ACS Catal.* **2017**, *7*, 8130–8133.

(46) Knappeová, B.; Mlýnský, V.; Pykal, M.; Šponer, J.; Banáš, P.; Otyepka, M.; Krepl, M. Comprehensive Assessment of Force-Field Performance in Molecular Dynamics Simulations of DNA/RNA Hybrid Duplexes. *J. Chem. Theory Comput.* **2024**, *20*, 6917–6929.

(47) Nakamura, T.; Zhao, Y.; Yamagata, Y.; Hua, Y.-j.; Yang, W. Watching DNA Polymerase η Make a Phosphodiester Bond. *Nature* **2012**, *487*, 196–201.

(48) Da, L.-T.; Wang, D.; Huang, X. Dynamics of Pyrophosphate Ion Release and Its Coupled Trigger Loop Motion from Closed to Open State in RNA Polymerase II. *J. Am. Chem. Soc.* **2012**, *134*, 2399–2406.

(49) Da, L.-T.; Pardo Avila, F.; Wang, D.; Huang, X. A Two-State Model for the Dynamics of the Pyrophosphate Ion Release in Bacterial RNA Polymerase. *PLoS Comput. Biol.* **2013**, *9*, e1003020.

(50) Da, L.-T.; E, C.; Duan, B.; Zhang, C.; Zhou, X.; Yu, J. A Jump-from-Cavity Pyrophosphate Ion Release Assisted by a Key Lysine Residue in T7 RNA Polymerase Transcription Elongation. *PLoS Comput. Biol.* **2015**, *11*, e1004624.

(51) Aho, N.; Groenhof, G.; Buslaev, P. Do All Paths Lead to Rome? How Reliable Is Umbrella Sampling Along a Single Path? *J. Chem. Theory Comput.* **2024**, *20*, 6674–6686.

(52) Baştuğ, T.; Chen, P.-C.; Patra, S. M.; Kuyucak, S. Potential of Mean Force Calculations of Ligand Binding to Ion Channels from Jarzynski's Equality and Umbrella Sampling. *J. Chem. Phys.* **2008**, *128*, 155104.

(53) Atis, M.; Johnson, K. A.; Elber, R. Pyrophosphate Release in the Protein HIV Reverse Transcriptase. *J. Phys. Chem. B* **2017**, *121*, 9557–9565.

(54) Kognole, A. A.; MacKerell, A. D. Mg^{2+} Impacts the Twister Ribozyme through Push-Pull Stabilization of Nonsequential Phosphate Pairs. *Biophys. J.* **2020**, *118*, 1424–1437.

(55) Buelens, F. P.; Leonov, H.; de Groot, B. L.; Grubmüller, H. ATP–Magnesium Coordination: Protein Structure-Based Force Field Evaluation and Corrections. *J. Chem. Theory Comput.* **2021**, *17*, 1922–1930.

(56) Allnér, O.; Nilsson, L.; Villa, A. Magnesium Ion–Water Coordination and Exchange in Biomolecular Simulations. *J. Chem. Theory Comput.* **2012**, *8*, 1493–1502.

(57) Panteva, M. T.; Giambasu, G. M.; York, D. M. Force Field for Mg^{2+}, Mn^{2+}, Zn^{2+}, and Cd^{2+} Ions That Have Balanced Interactions with Nucleic Acids. *J. Phys. Chem. B* **2015**, *119*, 15460–15470.

(58) Grotz, K. K.; Cruz-León, S.; Schwierz, N. Optimized Magnesium Force Field Parameters for Biomolecular Simulations with Accurate Solvation, Ion-Binding, and Water-Exchange Properties. *J. Chem. Theory Comput.* **2021**, *17*, 2530–2540.
